# Inter-brain synchrony in teams predicts collective performance

**DOI:** 10.1093/scan/nsaa135

**Published:** 2020-09-30

**Authors:** Diego A Reinero, Suzanne Dikker, Jay J Van Bavel

**Affiliations:** Department of Psychology, New York University, New York, NY, USA; Department of Psychology, New York University, New York, NY, USA; Department of Psychology, New York University, New York, NY, USA

**Keywords:** inter-brain synchrony, hyperscanning, collective performance, group identification, teams

## Abstract

Despite decades of research in economics and psychology attempting to identify ingredients that make up successful teams, neuroscientists have only just begun to study how multiple brains interact. Recent research has shown that people’s brain activity becomes synchronized with others’ (inter-brain synchrony) during social engagement. However, little is known as to whether inter-brain synchrony relates to collective behavior within teams. Here, we merge the nascent field of group neuroscience with the extant literature of team dynamics and collective performance. We recruited 174 participants in groups of 4 and randomly assigned them to complete a series of problem-solving tasks either independently or as a team, while simultaneously recording each person’s brain activity using an electroencephalography hyperscanning setup. This design allowed us to examine the relationship between group identification and inter-brain synchrony in explaining collective performance. As expected, teammates identified more strongly with one another, cooperated more on an economic game, and outperformed the average individual on most problem-solving tasks. Crucially, inter-brain synchrony, but not self-reported group identification, predicted collective performance among teams. These results suggest that inter-brain synchrony can be informative in understanding collective performance among teams where self-report measures may fail to capture behavior.

## Introduction

One of the distinctive features of humans is our ability to identify and cooperate with groups, and to leverage this ability for collective success. Decades of research has explored group dynamics, attempting to understand what makes teams successful (e.g. Kozlowski and Bell, 2003; Kozlowski and Ilgen, 2006). This research has identified factors such as a group’s shared identity (Hogg *et al.*, 2004; Hertel and Solansky, 2011), psychological safety (Edmondson, 1999), emotional intelligence (Druskat and Wolff, 2001; Jordan *et al.*, 2002) or a group’s collective intelligence, a latent factor that predicts collective performance across a range of tasks (Woolley *et al.*, 2010). Yet predicting team success has remained an area of ongoing research for both scientists (Cooke and Hilton, 2015)^1^ and industry leaders (e.g. see Google’s quest to identify markers of successful teams in which they were unable to find strong patterns; Duhigg, 2016).

Despite humans evolving and living in a world of groups and teams (Caporeal, 1997; Dunbar, 1998; Wuchty *et al.*, 2007), most previous work studying the neuroscience of human psychology has focused on individuals in isolation, responding to static images or words. As a result, scientists still know very little about how the brain supports dynamic group interactions. The study of real-world social exchanges was thus dubbed the ‘dark matter of social neuroscience’ (Schilbach *et al.*, 2013) and deemed ‘hot topics for future study’ (Stanley and Adolphs, 2013). In the current research, we target this gap and examine the psychology and neuroscience underlying collective performance and group cooperation.

Neuroscientists have recently emphasized the importance of studying how multiple brains interact (Hasson *et al.*, 2012; Redcay and Schilbach, 2019; Wheatley *et al.*, 2019) in more ecologically valid social contexts (Matusz *et al.*, 2019; Shamay-Tsoory and Mendelsohn, 2019) with more naturalistic stimuli (Sonkusare *et al.*, 2019). Incorporating these methods can shed light on the neural processes associated with dynamic social interactions and coordinated action. Research examining the brain-to-brain dynamics underlying social interactions across a variety of tasks (e.g. mimicry, joint button pressing, cooperative computer games, economic games, musical performances, shared attention, verbal communication; see Czeszumski *et al.*, 2020 for review) has revealed that people’s brain activity exhibits greater inter-brain connectivity with others during face-to-face interactions when there is joint action (e.g. Dumas *et al.*, 2010) or shared attention and a common goal (e.g. Dikker *et al.*, 2017). In keeping with much of the literature, we will use the term inter-brain synchrony to describe this phenomenon. Little is known, however, about how inter-brain synchrony is related to collective performance in groups.

Some research has found that cooperation increases inter-brain synchrony. For example, dyads show greater inter-brain synchrony when cooperating with a partner on a button pressing task than when working competitively (Cui *et al.*, 2012; Cheng *et al.*, 2015; Pan *et al.*, 2017; Reindl *et al.*, 2018) or independently (Funane *et al.*, 2011; Mu *et al.*, 2016 2017; Hu *et al.*, 2017).^2^ These effects are not fully explained by shared motor movements and are often concentrated in brain areas or frequencies associated with attention and mentalizing, suggesting that inter-brain synchrony may arise, at least in part, from the mutual recognition of a partner’s role and actions or perception-action coupling (Preston and De Waal, 2002; Sadato, 2017; Schippers and Keysers, 2011; though see, Jacob, 2009). Indeed, the social awareness and increase in inter-brain synchrony that arises when coordinating with a partner may reflect an aspect of how social facilitation impacts team performance (Szymanski *et al.*, 2017) and collaborative learning (Antonenko *et al.*, 2019). Taken together, inter-brain synchrony might reflect the sharing of attention or psychological states necessary for coordinating actions (Mu *et al.*, 2018; Minagawa *et al.*, 2018; Dikker *et al.*, 2019), which could facilitate collective performance.

Understanding collective performance requires moving beyond dyads to groups, where social identities and group processes (e.g. process loss, groupthink) are relevant. Identification with one’s group may activate different norms (Bicchieri, 2002) and may therefore influence group cooperation (Brewer and Kramer, 1986; De Cremer and Van Vugt, 1999; Pärnamets *et al.*, 2020). For instance, compared to dyads, groups exhibit different patterns of non-verbal behaviors (e.g. eye gaze) and communication (Solano and Dunnam, 1985; Herrera *et al.*, 2011), as well as different motivations to trust and cooperate with others (Zhou and Zhang, 2006; Wildschut and Insko, 2007; Pereda *et al.*, 2019). Indeed, groups of four may be the optimal group size for everyday collaborations as it maintains individual responsibility and efficacy (Kameda *et al.*, 1992), while enhancing collective action on behalf of a shared group identity (Baumeister *et al.*, 2016). Yet the role of inter-brain synchrony has rarely been studied beyond 1:1 interactions: only a handful of such studies investigate groups of three or more people simultaneously (Jiang *et al.*, 2015; Nozawa *et al.*, 2016; Dikker *et al.*, 2017; Müller *et al.*, 2018; Bevilacqua *et al.*, 2019) compared to over 100 studies and counting that investigate dyadic interactions. As such, there is a need to examine the relationship between social identification and inter-brain synchrony on collective performance in groups.

Recent methodological developments have afforded the opportunity to study brain activity during real collective decisions (Liu *et al.*, 2018). For instance, recent work used portable electroencephalography (EEG) headsets in a school classroom and found that student-to-group inter-brain synchrony was highest when students watched videos or engaged in group discussion, relative to when the teacher read aloud or lectured (Dikker *et al.*, 2017; Bevilacqua *et al.*, 2019). Moreover, student-to-group inter-brain synchrony was associated with individual differences in group affinity, trait empathy and social dynamics. In the present study, we applied the same methods to examine whether inter-brain synchrony predicts collective performance.

Specifically, we merged work on inter-brain synchrony with team dynamics and collective performance by using group problem-solving tasks shown to capture a group’s collective intelligence (Woolley *et al.*, 2010 2015; Engel *et al.*, 2014). While previous inter-brain synchrony research has used novel tasks (e.g. mental time counting and joint button pressing) during cooperative *vs* competitive conditions, our tasks are designed to assess collective performance directly: some tasks lend themselves to teamwork while others expose group fallibilities (e.g. process loss). Moreover, we included a control condition of groups who experience similar sensory input but who lacked the group cohesion and interdependency found among teammates, addressing a common critique of inter-brain synchrony research (Szymanski *et al.*, 2017). We also measured economic cooperation using a one-shot public goods game, which is an established decision-making task mimicking collaborative social dilemmas (Olson, 1965; Hardin, 1968). Using these collective performance and cooperation tasks allowed us to test whether inter-brain synchrony adds predictive validity for ecologically valid group decisions and outcomes above and beyond self-report measures (e.g. social identification). As such, our research builds on the brain-as-predictor approach, which has proven useful in predicting the success of health campaigns, consumer choices and responsiveness to therapy (Berkman and Falk, 2013).

### Overview

This research examines the role of inter-brain synchrony in collective performance. We studied cooperative and competitive groups and simultaneously recorded EEG from groups of participants as they completed a wide range of problem-solving tasks. We included groups where individuals worked on the same set of tasks as teams but did so independently and competitively as a control condition. Given previous research linking inter-brain synchrony to social coordination and cooperation, we hypothesized that if people are part of a team and there is group cohesion and interdependence between them to reach a shared goal that we would see higher inter-brain synchrony among cooperative teams relative to competing individuals. Moreover, we hypothesized that group identification and inter-brain synchrony would each predict collective performance: past research has shown that group identity is important for team success (e.g. Hogg *et al.*, 2004; Frings *et al.*, 2008; Hertel and Solansky, 2011), and inter-brain synchrony has been linked to shared attention and effective social coordination (e.g. Dikker *et al.*, 2017; Hu *et al.*, 2017). Finally, we examined if inter-brain synchrony would predict performance during collective decision-making over and above traditional self-report measures of group cohesion as well as behavioral indices of cooperation and empathic traits such as accurate emotion perception.

## Methods

### Participants

During the spring and fall of 2017, we recruited 174 undergraduates in groups of 4, forming 44 groups (*M*_age_ = 19 years, SD_age_ = 1.3 years, 70% female; see supplement Figure S1 for further demographic details),^3^ which well exceeds the median sample size for inter-brain synchrony studies (33 participants; Reinero, Unpublished manuscript). Our stopping rule was determined by recruiting as many groups as possible by the end of that fall academic semester. We formed groups based on scheduling availability, and as a result, groups could be either mixed-sex or same-sex.^4^ The majority of participants within a group were strangers, though a small fraction (approximately 9% of participants) were casual acquaintances because they had previously taken the same class. One group was excluded from inter-brain synchrony analyses due to noisy EEG data. All participants had normal or corrected-to-normal vision and no history of neurological or psychiatric disorders. Written informed consent was obtained from each participant prior to the experiment. The study procedures were approved by the University Committee on Activities Involving Human Subjects. We report how we determined our sample size, all data exclusions, all manipulations and all measures in the study. All data and materials are publicly available on OSF: https://osf.io/5u7hm/.

### Materials & procedure

#### Setup.

**Fig. 1.**
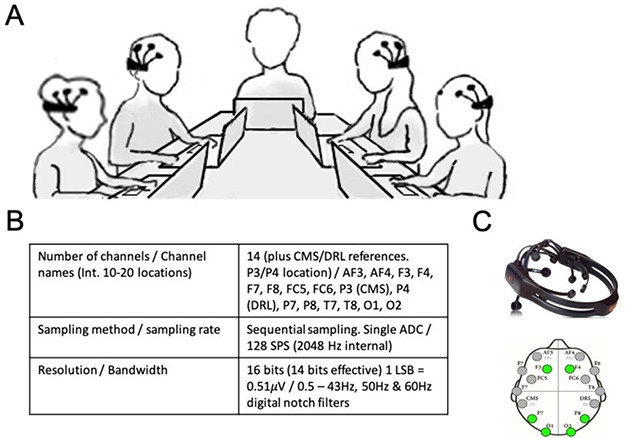
Testing room layout and EMOTIV EPOC headset/electrode array. Panel A is a sketch of the testing room layout with the experimenter at the head of the table and the four participants on either side. Panel B shows the hardware specifications of the EMOTIV EPOC EEG headset. Panel C shows the EMOTIV EPOC EEG headset and a top view of the electrode locations with those included in analysis marked in green (text).

Participants were instructed to wait in a common waiting area until all four people in their group arrived. Once the full group had arrived, participants were taken to our testing room and were seated across from each other at a rectangular table with a laptop in front of each participant (Figure 1A). We fit all participants with a 14-channel portable EMOTIV EPOC wireless EEG headset (Figure 1B for technical specifications; see Debener *et al.*, 2012; Dikker *et al.*, 2017, for validation). Signal quality and electrode connectivity were determined based on visual inspection using the EMOTIV’s TestBench software program (supplement Figure S2A). Although data quality from these portable and wireless EMOTIV EPOC EEG headsets may be lower compared to standard wired laboratory-grade equipment (Krigolson *et al.*, 2017), they have been used in other studies and proven suitable for hyperscanning and shown to detect meaningful inter-brain synchrony (e.g. Dikker *et al.*, 2017 2019; Bevilacqua *et al.*, 2019). Moreover, they offer important ecological validity benefits as they are less intrusive on a participant’s experience and allow for a more natural social interaction. As noted later in the Methods, we took various steps to ensure that our data met rigorous standards.

After EEG setup, we connected all four EEG headsets to a main computer in the testing room, which used custom software (developed in C++ and OpenFrameworks, see supplement Figure S2B for screenshot; https://openframeworks.cc/; see Dikker *et al.*, 2017; 2019; note that this software is only compatible with EPOC and not EPOC+) to record EEG data from all four headsets simultaneously. One experimenter, who was the same experimenter for all groups, remained in the testing room and oversaw the experiment (managing the simultaneous EEG recordings from the main computer and providing instructions to participants). All sessions were video- and audio-recorded.

**Fig. 2.**
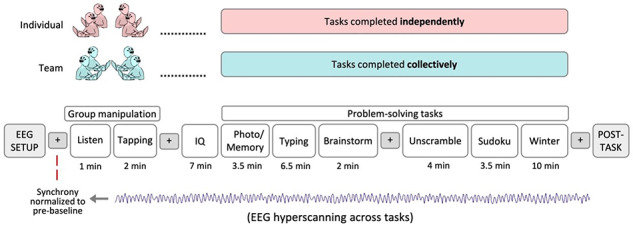
Experimental Procedure and Timing. Groups were randomly assigned to either the team or individual condition (group manipulation). Groups performed a hand-tapping exercise and then began their computerized problem-solving tasks. Teams completed these problem-solving tasks together while individuals completed these tasks individually. EEG was recorded from all four participants throughout the group manipulation and the problem-solving tasks. Interleaved throughout the experiment were four 2 minute resting periods where participants focused their attention on a fixation cross on their computer screen (indicated by the + sign in the figure and referred to as baselines).

After recording EEG for the initial resting period, we asked participants to introduce themselves to one another. Participants were asked to share their name, year in school, where they lived and their major or intended major. At this point, we manipulated group cohesion and interdependency by randomly assigning the group to either the Team (*N* = 87; 22 groups) or Individual (*N* = 87; 22 groups) condition. As noted previously, we included groups of individuals as a control condition as they would experience similar sensory input (working on the same set of time-locked tasks) but would lack the group cohesion and interdependency found among teammates. Figure 2 outlines the experimental procedure and is described further below.

### Group manipulation

#### Team condition.

we sought to enhance group cohesion and interdependency by telling groups in the team condition that they would be working together and competing against other teams on a series of problem-solving tasks. Rewards were contingent on overall team performance ($200 bonus split equally—$50/person—if their team was ranked in the top 5% of teams). We also asked teams to generate a team name (the experimenter left the room for 2 minutes during this time to allow teammates to bond amongst themselves and feel more at ease), which was written on a name card and placed in the center of the table. Immediately afterward, teams performed a hand-tapping exercise while facing each other, where they tried to synchronize their hand tapping to an instrumental beat (listen here: https://osf.io/cf7bj/) that played from the experimenter’s computer. Specifically, we played an instrumental beat and asked participants to simply listen for 1 min to familiarize themselves with the beat (‘Listen’ in Figure 2). Then we restarted the instrumental track and this time the team attempted to tap to the beat together with the instrumental track as best they could. Teammates faced each other, focused their attention at their team’s name card in the center and tapped on the table with their hand that was nearest the middle. After the instrumental track ended, teammates continued to tap the beat for one more minute without the assistance of the instrumental track (‘Tapping’ in Figure 2). Such physical synchrony and hand-tapping exercises have previously been shown to increase feelings of similarity and rapport (Macrae *et al.*, 2008; Valdesolo and DeSteno, 2011; Nozawa *et al.*, 2019). All of these procedures were done with the goal of activating a shared social identity and enhancing group cohesion and interdependency.

#### Individual condition.

In contrast, we removed group cohesion and interdependency in the individual condition by telling the group of individuals that they would be working individually and competing against each other and others in the study. Rewards were contingent on individual performance ($50 if they ranked in the top 5% of individuals; thus, keeping the monetary incentive per person the same across conditions). We asked individuals to generate a personal code name (the experimenter again left the room during this time), which was written on a name card and placed in front of each individual. Individuals also performed the same hand-tapping exercise as teams, but they did so while facing away from their competitors and focusing on their own hand-tapping (i.e. no coordination with others required). Specifically, each individual turned their chair such that they were sitting back-to-back with the person that was next to them. This limited their ability to maintain any eye contact or engage in other forms of social interaction with their competitors. Each individual then tapped the instrumental beat softly on their own leg, which was beneath the table and out of sight of their competitors. This allowed each person to focus on their own hand-tapping and not be distracted by the sound of tapping done by others. All of these procedures were done with the goal of activating an individual identity and fracturing group cohesion and interdependency, while still maintaining a similar procedure between the team and individual condition.

### Problem-solving tasks

EEG data were recorded from all four participants simultaneously during the hand-tapping exercise and during all subsequent tasks. After completing the group manipulation, all participants individually completed an abbreviated IQ test on their laptops (Raven’s Advanced Progressive Matrices; a non-verbal test of abstract reasoning). This was to ensure our use of random assignment was successful, such that the IQ of participants was evenly distributed across each group and was not a confound when testing collective performance.^5^ Afterward, participants used their laptops to complete a series of online problem-solving tasks previously used to calculate a measure of collective intelligence (Woolley *et al.*, 2010).

Tasks ranged across the McGrath Task Circumplex (Straus, 1999) in terms of skills required to complete them (e.g. Generating, Choosing, Negotiation and Executing), and included the following tasks in this order: answering questions about a photograph from memory (Photo/Memory; 3.5 min), typing a passage of text (Typing; 6.5 min), brainstorming creative uses of a brick (Brainstorm; 2 min), unscrambling words (Unscramble; 4 min), completing a sudoku puzzle (Sudoku; 3.5 min) and rank-ordering items needed for survival in a wintery plane crash scenario (Winter; 10 min). See supplement ‘Section 3: Task descriptions and scoring’ for detailed descriptions of each task. Some tasks lend themselves to teamwork more so than others. For example, brainstorming creative uses of brick likely benefit from teamwork whereas typing a passage of text is much easier as an individual as you do not have to waste time coordinating with others, and are not distracted by the real-time writing of others.

**Fig. 3.**
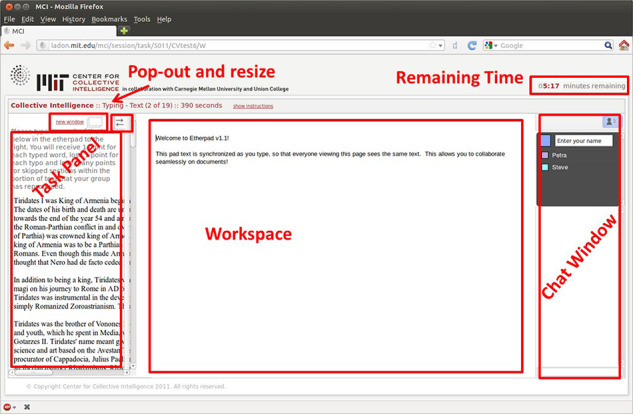
Example screenshot of online system used to administer problem-solving tasks. The right-hand chat window allowed teammates to communicate (for competing individuals, the chat was disabled and was simply a ‘think-aloud’ window). The middle task panel is where answers could be entered. The left-hand instructions panel contained instructions and other stimulus materials for the task. Time remaining for a given task was displayed in the top right corner.

Participants in the team condition completed these problem-solving tasks together in a shared document where each teammate’s edits could be seen in real-time by all team members. Although participants were not allowed to talk out loud (to avoid additional noise in the EEG data), participants in the team condition were able to communicate with their teammates via an online chat built into the online task system. In contrast, participants in the individual condition completed each problem-solving task individually. The online chat was disabled for individuals, though they were told they could use that text-box space as a ‘think-aloud’ tool to jot down any thoughts or ideas they had while working on the problem-solving tasks. Figure 3 shows an example team condition screenshot from the online system used to administer the tasks.

The online system guided participants through the tasks together, ensuring that all participants worked on the same tasks at the same time in the same order, regardless of whether they were working as a team or independently. This was done in an attempt to standardize the sensory input for participants across conditions, such that the key difference between the conditions was a shift in the psychological experience from one of a collective team (social identity) to one of a competitive individual (personal identity). In this way, our individual condition acted as a control group, as participants in this condition would be doing the same tasks at the same time—matching any possible entrainment to a shared stimulus that teams might experience—but without any social coordination.

Each problem-solving task had a fixed duration (ranging between 2 and 10 min; see Figure 2), after which the next task would automatically begin. The entire battery of problem-solving tasks took 36.5 min to complete. In addition, participants engaged in four 2 min resting periods where they focused their attention on a fixation cross in the middle of their laptops (indicated by the fixation crosses in Figure 2) while EEG was recorded. These resting periods occurred at the beginning of the experiment (pre-baseline), after the group manipulation (post-baseline), midway through the problem-solving tasks (mid-baseline) and at the end of the problem-solving tasks (end-baseline). After the problem-solving tasks, EEG headsets were removed, and participants completed several post-task measures on their laptops.

### Post-task measures

*Cooperation*: The first measure was a one-shot public goods game, which is a common measure of cooperation (Hamburger, 1973; Fox and Guyer, 1978; Wills *et al.*, 2018). We reminded participants that if they outperformed others (either as a team in the team condition or as an individual in the individual condition) that they would receive a $50 bonus personally, and we asked them to consider how they would handle $10 of those $50. Thus, all participants were ‘endowed’ with $10. Each participant could contribute any amount (or none) of their $10 to their group’s public pot. The total amount in the public pot would then be doubled and equally re-distributed to each participant, regardless of whether or not that participant contributed. As such, there is an incentive to be selfish and hope to reap the rewards of generous others. However, if everyone acts selfishly, the group forgoes the opportunity to increase their collective earnings. If instead everyone was prosocial and contributed all $10 to the pot, participants could double their earnings to $20 (four players contributing $10 would put $40 in the pot, which would then be doubled to $80, and then equally re-distributed to the four players such that each would receive $20). See supplement ‘Section 2: Additional items’ for further methodological details.

*Group Identification and Groupiness*: Next, we measured participants’ group identification using a 3-item measure (I value this group; I like this group; I feel connected to this group; Van Bavel and Cunningham, 2012) where each item was on a 100-point scale going from 0 = strongly disagree to 100 = strongly agree. The reliability of this scale for our data was quite strong (a = 0.88) and participants’ overall scores, across conditions, were as follows: *M* = 60.98, SD = 22.29. We also measured participants’ trait disposition toward groups in general, called groupiness, using a 12-item measure (Dunham & Van Bavel, unpublished). Example items include*, ‘An important part of my identity is being a part of some group’; ‘the social groups we belong to are one of the most important things in our lives’; ‘I am happiest when I am on my own’* (reverse-scored), and participants made ratings on a 7-point Likert scale that went from 1 = strongly disagree, 2 = disagree, 3 = somewhat disagree, 4 = neither agree nor disagree, 5 = somewhat agree, 6 = agree, 7 = strongly agree. The reliability of this scale for our data was satisfactory (*a* = 0.71) and participants’ overall scores, across conditions, were as follows: *M* = 4.11, SD = 0.69.

*Emotion Perception*: Given past research suggesting that inter-brain synchrony may be related to attentional focus and empathic processes (e.g. mentalizing), we measured participants’ emotion perception abilities using the Reading the Mind in the Eyes Test (Baron-Cohen *et al.*, 2001). In this test, participants are shown the eye region of a person’s face and must determine which emotion that person is feeling by selecting among a set of four emotion options. Participants completed 36 trials, with higher scores indicating more accurate emotion perception (*M = *27.3, SD = 3.6). Our Reading the Mind in the Eyes Test results are consistent with the student sample data (*N* = 103) in Baron-Cohen et al., (Baron-Cohen et al., 2001*; M = 28.0*, SD* = 3.5).*

*Demographics & Debriefing*: We then collected demographics including sex, race, age, political orientation, religiosity and English proficiency. Afterward, we briefly interviewed each participant privately to ask how they felt the study went, what they liked/disliked and if they knew anyone from the group previously (and if so, what the nature of their relationship was). We then debriefed and thanked participants as a group.

### EEG pre-processing

EEG data were recorded from 44 groups of 4 subjects (*N* = 174) across 14 separate tasks each, totaling 47.5 min per subject (Figure 2). EEG data pre-processing was identical to Dikker *et al.* (2019).

At acquisition, a double notch filter at 50 and 60 Hz was applied, which essentially renders any frequency response above 43 Hz uninterpretable. Data were further demeaned using the ft_pre-processing function in the Fieldtrip toolbox (Oostenveld *et al.*, 2011), and high-pass filtered at 0.5 Hz prior to time-frequency analysis to remove slow fluctuations. Datasets with intermittent data loss, data repetition or misalignment between the four headsets were flagged and excluded from further processing. We next set out to identify physiological and hardware artifacts. After removing 50 ms before and 300 ms after instances in the EEG data that were flagged by the EMOTIV algorithms as containing blinks and vertical/horizontal eye movements, we used the Fieldtrip toolbox (Oostenveld *et al.*, 2011) to remove data instances with Signal Jumps, EOG-like Artifacts, Clipping Artifacts and Head Movements (the latter were removed using information from a 2-axis gyroscope built into the EEG headset). Research assistants then manually inspected the data to identify possible noisy channels. They used EEGLAB (Delorme and Makeig, 2004) to manually scroll through the continuous EEG data for each dataset, noting any channels that appeared flat or drifting (usually representing poor scalp connection). Any channel that was flat or drifting for >33% of the time was flagged as ‘bad’. They also visually inspected the spectral plots for each dataset, noting any channels that appeared abnormal. Finally, they also utilized EEGLAB’s auto-channel rejection to note any channels that exceeded a kurtosis *z*-score threshold of 3 s.d. There was often overlap between the ‘bad’ channels identified via these four metrics (visual flat, visual drifting, visual spectral plot and auto-channel rejection flag). Any channel that was flagged by two or more of these criteria was considered ‘bad’ and was removed from further analysis.

The remaining raw data were segmented into 1 s epochs. Each epoch that overlapped with bad segments as defined through the procedure described above was removed. Tasks for which less than 30 s of data remained after this step were removed from further analysis. These pre-processing steps were employed for each task for each participant and resulted in the preservation of an average of 11 of 14 tasks per group in both the individual and team condition. While there was some data loss, there was no significant difference in data loss between the team and individual condition across tasks, *t*(18.94) = 0.82, *P* = 0.421 (supplement ‘Section 5: Group inter-brain synchrony data loss’, Table S2, and Figure S5). One group in the individual condition was removed during EEG pre-processing.

### Time-frequency and inter-brain synchrony analysis

Data from each participant were concatenated for the time-frequency analysis, which was performed using a Hanning-taper transformation (Maris and Oostenveld, 2007) on each time point of each cleaned dataset with a window length of five periods of each frequency (1–40 Hz). Inter-brain synchrony was quantified following a similar rationale as our previous group synchrony work (Dikker *et al.*, 2017; Bevilacqua *et al.*, 2019), using the same computations as Dikker *et al.* (2019).

Coherence was computed over the spectral coefficients from each paired electrode, between each pair of participants in a group, for each ‘overlapping’ 1 s epoch (i.e. EEG data were preserved for both headsets for that particular time segment), according to the following procedure: first, the auto spectral density (power) was computed (*S_xx_* (*f*) and *S_yy_* (*f*) in equation (1.1 and 1.2)) over the time-frequency spectral coefficients series *X*(*t, f*) and *Y*(*t, f*) of two separate EEG channels, after which the cross-spectral density between them was derived (*S_xy_* (*f*) in equation (1.3)). Then, coherence (equation (1.5)) was computed as the absolute value of coherency equation (1.4)), a complex number C_xy_ (f), for which the phase indicates the average phase difference between the two series and the magnitude indicates how consistent this phase difference is.

(1.1) \begin{equation*} {S_{xx}}\left(\, f \,\right) = \mathop \sum \limits_{t = 1}^N *X\left( {t,f} \,\right) \cdot {X^*}\left( {t,f} \,\right)\end{equation*}

(1.2) \begin{equation*} {S_{yy}}\left(\, f \,\right) = \mathop \sum \limits_{t = 1}^N *Y\left( {t,f} \,\right) \cdot {Y^*}\left( {t,f} \,\right)\end{equation*}

(1.3) \begin{equation*} {S_{xy}}\left(\, f \,\right) = \mathop \sum \limits_{t = 1}^N *X\left( {t,f} \,\right) \cdot {Y^*}\left( {t,f} \,\right)\end{equation*}

(1.4) \begin{equation*} {C_{xy}}\left(\, f \,\right) = {{{S_{xy}}\left(\, f \,\right)} \over {{{\left( {{S_{xx}}\left(\, f \,\right) \cdot {S_{yy}}\left(\, f \,\right)} \right)}^{1/2}}}}\end{equation*}

(1.5) \begin{equation*} Coh = \left| {{C_{xy}}\left(\, f \,\right)} \right|\end{equation*}

This resulted in one coherence value per frequency and electrode pair for each subject pair within the group, in each task. To match the inter-brain synchrony approach used in Dikker *et al.* (2017) and Bevilacqua *et al.* (2019), we then averaged coherence values across 1–20 Hz over 6 electrode pairs of interest (‘F3’ ‘F4’ ‘O1’ ‘O2’ ‘P7’ ‘P8’; see Figure 1C), after removing bad channels that were identified using the visual inspection procedure described above. These six electrodes were previously established to be most likely to exhibit decent data quality (Figure 1C; Dikker *et al.*, 2017), and focusing on these channels may help circumvent large discrepancies between groups with respect to the number of channels that were considered for analysis (due to differences in the number of channels considered for analysis after removing bad channels). For this same reason, we focused on pairwise comparisons rather than computing synchrony between all possible electrode combinations between pairs: While many prior studies have found that behaviorally relevant inter-brain synchrony need not be limited to pairwise scalp locations across subjects (e.g. Dumas *et al.*, 2010), in our case (i) electrode placement may vary across participants (e.g. Dikker *et al.*, 2017), and (ii) there was some variation between subjects in terms of which channels produced usable data and which produced noisy data for a given task. This renders any sensor-specific effects largely uninterpretable.

Pairwise inter-brain synchrony values were then averaged across the group, providing a measure of group inter-brain synchrony for each task. As absolute synchrony values may depend on many idiosyncratic factors, all analyses were conducted over each group’s percent coherence change from baseline (the pre-baseline task). To avoid undue influence of outliers, we excluded any normalized group inter-brain synchrony values that exceeded 3 s.d. from the mean (less than 1% of synchrony values were excluded). The results are robust to this outlier exclusion.

## Results

Given the design of our experiment, data existed at the subject and group level. Individual difference measures of cooperation, group identification, emotion perception and demographics were at the subject level (each subject has their own data point; *N* = 174 participants). For some analyses, we obtain a group average for these individual differences (e.g. average group identification). Measures of inter-brain synchrony and performance were at the group level,^6^ with a data point for each task (*N* ≈ 473 for inter-brain synchrony; *N* = 308 for performance).^7^ All analyses match the level of variables, and we perform mixed models nesting by group for any analyses that include group level variables.

### Behavioral results

We first examined whether participants’ IQ significantly differed between the team and individual condition, to ensure it was not a confound when later examining collective performance. As expected, there was no difference in the proportion of correct answers on this test for participants in the team condition (*M *= 0.30, SD = 0.10) compared to the individual condition (*M *= 0.31, SD = 0.09), *t*(170.38) = −0.55, *P* = 0.579, suggesting that random assignment was successful.

**Fig. 4.**
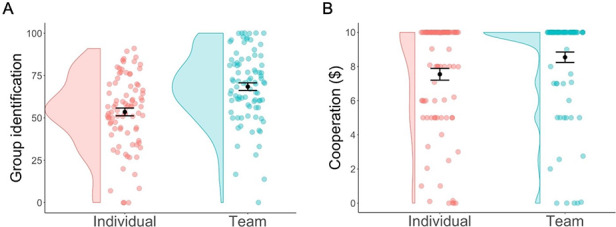
Panel A: teams (turquoise) were more highly identified than individuals (red). Panel B: teams cooperated more than individuals. Each dot represents a participant’s level of group identification (Panel A) or contribution to the public pot (Panel B). Means are indicated by a black dot with error bars representing +/− 1 standard error.

On the other hand, we expected participants who were part of a team and whose later performance was interdependent with their teammates, to be more highly identified with their group than participants who had competed against one another. Our manipulation appeared successful. First, participants in the team condition were more highly identified (*M* = 68.4, SD = 20.9) than participants in the individual condition (*M* = 53.5, SD = 21.2), *t*(171.97) = 4.68, *P*<0.001, *d* = 0.71 (Figure 4A). Second, our manipulation increased cooperation: participants in the team condition contributed more money to their group’s public pot (*M* = $8.54, SD = 2.86) than participants in the individual condition (*M* = $7.54, SD = 3.21; see Figure 4B), *t*(169.79) = 2.15, *P* = 0.033, *d* = 0.33. As can be seen from Figure 4B, most participants were highly cooperative, contributing the full $10 to their group’s public pot, but a difference emerged between conditions with 74% of participants in the team condition contributing the full amount compared to only 51% of participants in the individual condition. Thus, teammates were more cooperative, and this finding is notable given that the rational choice in a one-shot public goods game is to be selfish and not contribute to the public pot (e.g. there are no future-oriented reputational concerns as it is a one-shot dilemma, and any teams have already completed their joint work). There was no significant interaction between group identification and condition when predicting cooperation (*P* = 0.883).

**Fig. 5.**
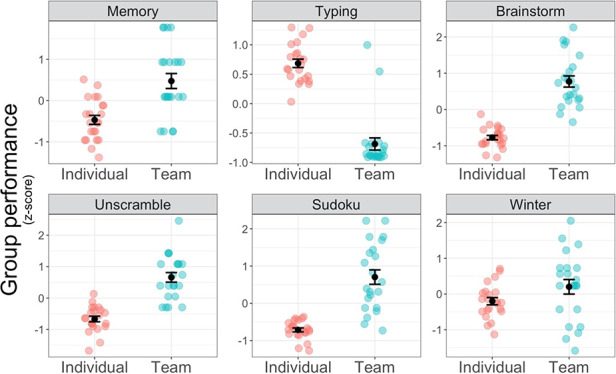
Teams (turquoise) outperformed the average of individuals within a group (red) across all six problem-solving tasks except the typing task which is prone to process loss. Each dot represents a group. Means are indicated by a black dot with error bars representing +/− 1 standard error.

We next examined team *vs* individual performance on the set of problem-solving tasks. We again found predicted differences between conditions, with teams outperforming individuals. Performing a linear mixed model estimating a random intercept for each group with condition predicting z-scored performance across the six main problem-solving tasks (excluding IQ as that task was done individually by all participants), teams performed much better on average, *b* = 0.71, SE = 0.09, *P*<0.001, 95% CI [0.52, 0.90]. As seen in Figure 5, teams significantly out-performed individuals on the memory task, *t*(34.37) = 4.46, *P*<0.001, brainstorming creative uses of a brick, *t*(27.36) = 9.41, *P*<0.001, unscrambling words, *t*(33.79) = 7.53, *P*<0.001, and sudoku, *t*(24.08) = 7.06, *P*<0.001, with a marginally significant trend for the winter survival task, *t*(30.84) = 1.77, *P* = 0.087. This was expected, as ‘many hands make light work’ and four teammates should outperform a single individual (Hill, 1982). However, teams suffer when they encounter a task prone to process loss or groupthink (Janis, 1982). Fortunately, we selected one of our tasks to provide an instance of process loss and individuals outperformed teams on this task, *t*(37.24) = −10.93, *P*<0.001 (‘Typing’ in Figure 5, top row, middle panel), providing discriminant validity.

### EEG results

**Fig. 6.**
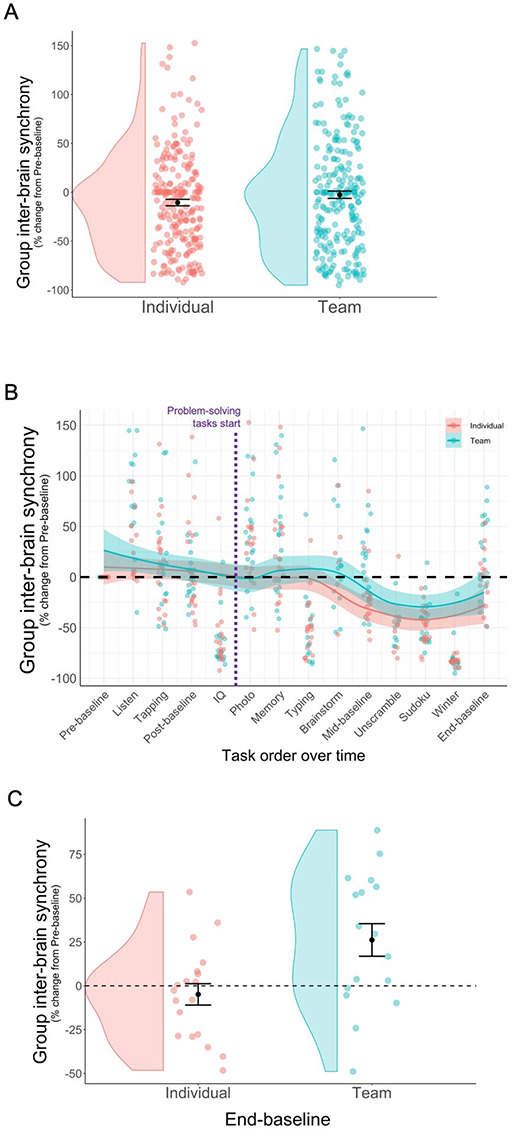
Panel A: group inter-brain synchrony was similar between teams (turquoise) and individuals (red) including all tasks with available EEG data. Panel B: group inter-brain synchrony decreased over time, though as seen in Panel C, this decrease was larger for individuals than teams by end-baseline. The y-axis represents each group’s percent coherence change from pre-baseline, with each dot representing a group inter-brain synchrony value for a given task. Means (Panel A) are indicated by a black dot with error bars representing +/− 1 standard error. The black dashed horizontal line (Panel B and C) represents zero change from pre-baseline.

Given past work linking inter-brain synchrony to cooperation and coordination with social partners, we predicted that inter-brain synchrony would be higher among teammates than a group of competing individuals. We conducted a linear mixed model estimating a random intercept for each group and used condition to predict group inter-brain synchrony across tasks. As seen in Figure 6A, we did not find a difference in group inter-brain synchrony between teams or individuals after correcting for baseline differences between conditions, *b* = 7.33, SE = 7.43, *P* = 0.329, 95% CI [−7.23, 21.89], N-S-J pseudo *R*^2^ = 12.42%.^8^ It is important to note in this regard that when such a baseline correction was *not* applied, individuals showed higher inter-brain synchrony than teams overall, but as can be seen most clearly in supplemental Figure S6B, this difference was mostly driven by differences in synchrony pre-manipulation, which cannot be attributed to group assignment. This drives home the well-documented caution that should be taken when interpreting between-subject designs.

In addition, although we manipulated group cohesion and interdependency between the conditions, we found no evidence that self-reported group identification was associated with group inter-brain synchrony, *b* = 0.47, SE = 0.48, *P* = 0.335, 95% CI [−0.47, 1.40], N-S-J pseudo *R*^2^ = 1.49%. Moreover, neither group emotion perception, *b =* 2.14, SE = 2.28, *P* = 0.354, 95% CI [−2.33, 6.62], nor group cooperation, *b =* 3.67, SE = 3.77, *P* = 0.336, 95% CI [−3.72, 11.06] (nor their interactions with condition), predicted group inter-brain synchrony.^9^ As such it appears, group inter-brain synchrony is not explained by individual measures of group identification, emotion perception or cooperation.

Using similar linear mixed models, we next examined whether inter-brain synchrony varied over time as a function of condition. Interestingly, as seen in Figure 6B and supplemental Figure S6B, group inter-brain synchrony decreased over time for both teams and individuals, *b* = −4.38, SE = 0.77, *P*<0.001, 95% CI [−5.89, −2.87]. Further, although there was no significant interaction between time and condition, individuals showed more inter-brain synchrony decrease than teams between pre-baseline and end-baseline *t*(28.14) = 2.79, *P* = 0.009 (Figure 6C). Indeed, the difference in inter-brain synchrony occurs for the team condition once they begin collective problem-solving (photo/memory task).

**Fig. 7.**
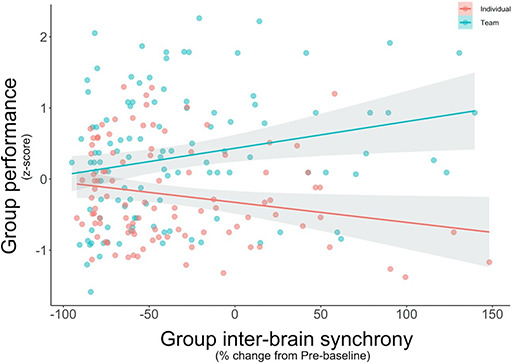
Group inter-brain synchrony predicted performance for teams (turquoise) but not for individuals (red). Synchrony values represent each group’s percent coherence change from baseline (the pre-baseline task), and each dot represents a group and a given task. Shading represents 95% confidence interval.

Next, we examined whether inter-brain synchrony was related to collective performance. We conducted a linear mixed model estimating a random intercept for each group using group inter-brain synchrony and condition to predict collective performance across tasks. In addition to the main effect of condition, with teams outperforming individuals, we found a significant interaction such that group inter-brain synchrony predicted performance among teams but not individuals, *b* = 0.35, SE = 0.11, *P* = 0.002, 95% CI [0.13, 0.56], N-S-J pseudo *R*^2^ = 14.46% (Figure 7; full model results in supplement, Table S3 step 1). Specifically, for every 1 s.d. increase in the percent coherence change from pre-baseline, teams did 0.20 s.d. better on performance relative to individuals. Thus, teams that showed higher inter-brain synchrony also performed better on average.

To ensure this association was not spurious, we randomly shuffled the inter-brain synchrony data and repeated the mixed model. Using the shuffled data, the interaction was no longer significant (*P* = 0.442), suggesting that the original association was not spurious. In addition, when we removed the process-loss task (typing) in an attempt to disaggregate tasks that benefited from teamwork from those that suffered from it, the interaction between inter-brain synchrony and condition on performance remained significant, *b* = 0.27, SE = 0.11, *P* = 0.014, 95% CI [0.06, 0.48]. This suggests this finding was relatively robust to how we specified collective performance.

In addition, the extant literature on team performance suggests that one important factor that contributes to team success is a group’s shared identity (Hogg *et al.*, 2004). As such, we next added self-reported group identification as an additional predictor in our mixed model, including its interaction with condition. Surprisingly, we found no evidence that self-reported group identification (nor its interaction with condition) predicted collective performance, *b* = 0.00, SE = 0.01, *P* = 0.600, 95% CI [−0.01, 0.02], N-S-J pseudo *R*^2^ = 14.68%, whereas group inter-brain synchrony continued to predict collective performance for teams, *b* = 0.35, SE = 0.11, *P* = 0.002, 95% CI [0.14, 0.57] (full model results in supplement, Table S3 step 2). In other words, inter-brain synchrony predicted performance among teams over and above participants’ self-report of how much they liked or valued their group.

Moreover, given that cooperation and emotional intelligence have been previously associated with team performance, we also added our post-task measures of cooperation and emotion perception to our mixed model as predictors, including their interactions with condition. Neither cooperation, *b* = 0.00, SE = 0.06, *P* = 0.986, 95% CI [−0.11, 0.11] nor emotion perception, *b* = 0.00, SE = 0.03, *P* = 0.987, 95% CI [−0.07, 0.07], N-S-J pseudo *R*^2^ = 14.63% (nor their interactions with condition), significantly predicted collective performance, whereas group inter-brain synchrony again continued to predict collective performance for teams (full model results in supplement, Table S3 step 3). Thus, when attempting to predict collective performance among teams using both self-report and behavioral indices, the best predictor was inter-brain synchrony.

## Discussion

Although humans are fundamentally social and work in teams nearly every day, little is known about the neural dynamics underlying such group interactions. Here, we found that teams were more highly identified, more cooperative and outperformed a group of individuals on tasks that avoided process loss. Critically, inter-brain synchrony predicted collective performance among teams, but not within members of a group who were working on the same tasks individually. Moreover, we found that inter-brain synchrony predicted collective performance among teams over and above self-reported group identification and behavioral indices of cooperation and emotion perception. This suggests that inter-brain synchrony can be informative in understanding collective performance among teams where self-report measures may fail to capture behavior.

Although the precise significance of inter-brain synchrony is still an active area of research, our results suggest that inter-brain synchrony may provide a useful implicit, unobtrusive and continuous measure that captures important aspects of interpersonal interactions. Specifically, we found that inter-brain synchrony predicted collective performance among teams but not among groups of individuals. This finding is related to results from our prior classroom group studies where inter-brain synchrony was predictive of social closeness between the student and their teacher as the teacher lectured but not while students were watching videos (i.e. when the teacher was co-present but not socially engaged with students; Dikker *et al.*, 2017). In the current research, inter-brain synchrony appears to better predict collective action rather than the psychological experience of being in a group. For example, inter-brain synchrony was significantly associated with collective performance but not self-reported group identification. Moreover, although inter-brain synchrony decreased over time in both conditions, this decrease was slightly buffered once collective problem-solving began in the team condition (i.e. the photo/memory task; see Figure 6B and supplemental Figure S6B). By the end of the study, teams were higher than individuals on synchrony (Figure 6C). This is consistent with previous work which found that inter-brain synchrony and focus decreased over time, and that such a decrease can be attenuated by socially relevant manipulations (Dikker *et al.*, 2019). This suggests that inter-brain synchrony may capture socially relevant neural information processing and may reflect or promote coordinated attention and action.

Moreover, some groups may be more coordinated than others. From anecdotal accounts based on the chat logs and post-study interviews with the participants, some teams had more group structure and leadership compared to others, which could track inter-brain synchrony. Thus, while inter-brain synchrony is relational, it is possible that inter-brain synchrony may be an implicit marker that identifies which members of a team are skilled at drawing the attention of others in a coordinated and persuasive manner. This is based on previous approaches which find that a common stimulus—such as a leader among a group—can be a synchronizer.

Indeed, previous research has found that measures of physiological synchrony (‘linkage’) may track who draws the attention of others within a group and is thus a persuasive leader (Thorson *et al.*, 2019). Leader–follower dynamics can emerge in more complex social exchanges, and inter-brain synchrony in the left TPJ (an area associated with mentalizing) has been found preferentially for leader–follower relationships (relative to follower–follower relationships) and is associated with leader’s communication skills and competence (Jiang *et al.*, 2015). These results suggest that effective leaders may be able to entrain others to their communications, which can be tracked in real-time through implicit measures such as neurophysiological synchronization. One speculative interpretation for our finding that teams did not have higher inter-brain synchrony than individuals, yet inter-brain synchrony tracked performance for teams, could be that shared entrainment to a stimulus (the time-locked computer tasks present in both conditions) may provide a basis for inter-brain synchrony, which can then be either diminished among uncoordinated teams (e.g. vis-à-vis attentional distractions or disengagement such as social loafing, Latané *et al.*, 1979) or enhanced among coordinated teams However, we are unable to formally corroborate this from our data, and future research should test these possibilities more directly.

Importantly, we found that inter-brain synchrony predicted collective performance above and beyond self-report measures of group identification and behavioral indices of cooperation and emotion perception. This finding points to the added value of inter-brain synchrony as an implicit measure that can predict team success. Although we believe replications and future research are needed to build confidence in the results, it is possible that inter-brain synchrony could inform latent aspects of collective performance, not visible through the myriad explicit individual difference measures or team structures that researchers typically collect when attempting to predict successful teams. For example, Google was unable to predict their best teams despite using a battery of demographic and personality measures (e.g. how frequently teammates socialized outside the office, if they had the same hobbies, if their educational backgrounds were similar, if their personalities were more extroverted or introverted, if gender composition mattered, etc, see Duhigg, 2016). Our work is in keeping with the brain-as-predictor framework: past work has shown that brain responses may be better predictors of smoking cessation relative to self-report measures alone (Falk *et al.*, 2011). Here, we similarly find that inter-brain synchrony may be able to predict which teams will be successful better than self-reported group identification.

### Limitations

We originally designed our experiment—and chose our specific set of problem-solving tasks—with the aim of testing whether inter-brain synchrony was associated with a group’s collective intelligence—a latent factor that was shown to predict a group’s performance across a range of tasks (Woolley *et al.*, 2010). However, we were unable to compute collective intelligence since the correlations between each task were not always positive (supplement Figure S4A/B), which was a prerequisite for computing this factor. We therefore computed a mean score on collective performance. Exploratory analyses also revealed that collective performance’s relationship with inter-brain synchrony varied across tasks (supplement Figure S7C). Moreover, recent research found that collective intelligence may be primarily explained by the summation of individual member’s IQ (Bates and Gupta, 2017), raising questions around the construct of collective intelligence. As such, more work should be done to validate collective intelligence.

In addition, in keeping with our previous work (Dikker *et al.*, 2017; Bevilacqua *et al.*, 2019), we calculated inter-brain synchrony using only six electrodes and only between identical electrode pairs, which limits our ability to determine the neural mechanisms of collective behavior. While there is no a priori reason to assume that inter-brain synchrony should be concentrated in the same electrode pairs, and that in fact inter-brain connectivity between different electrodes can be stronger and in fact more meaningful, it is important to note that for the headsets used in this study, electrode placement may vary considerably between individuals. Therefore, the locations are not to be interpreted.

Further, while we frame inter-brain synchrony as ‘predicting’ collective performance, our correlational results do not rule out the possibility that the causal direction is reversed (i.e. collective performance predicting inter-brain synchrony). In fact, it is possible that both causal pathways may operate in a dynamic fashion or that inter-brain synchrony acts as a passive indicator of coupling at the psychological and behavioral level. Unraveling these processes is a fruitful area for future research.

## Conclusion

Little is known regarding the neural dynamics associated with group interactions and collective performance. As groups continue to shape how we achieve our goals in fields ranging from education (e.g. classrooms) to politics (e.g. parties) to business (e.g. corporate teams), a gap exists between how people interact in the real-world and our understanding of the neural processes during group interactions. The present work aimed to help address that gap. We found that teams were more highly identified, more cooperative and out-performed individuals on tasks that avoided process loss. Moreover, our results suggest that inter-brain synchrony may predict successful teams where self-report measures and behavioral indices may fail to capture behavior. This represents a step towards understanding the neuroscience and psychology underlying collective performance and cooperation.

## Supplementary Material

nsaa135_Supp

## Footnotes

1 The 2015 report from the National Academy of Sciences studied how to enhance the effectiveness of collaborative research in science teams (Cooke and Hilton, 2015). According to some, it has been one of their most downloaded reports in history.

2 When cooperating on these button pressing tasks, participants are attempting to minimize the time difference between their respective button presses. When competing, participants are attempting to press the button faster than their partner. Note, some research has found inter-brain synchrony during competition as well, see Liu *et al.* (2015) and Liu *et al.* (2017).

3 Due to last minute no-shows, two of our groups consisted of 3 people instead of 4.

4 Reflecting our subject pool’s population of undergraduates within the psychology department, the majority of groups were either all female or mostly female: 11 groups were all female, 18 groups had three females and one male, 1 group had two females and one male, 8 groups had two females and two males, and 6 groups had one female and 3 males.

5 All groups completed the IQ test (an individual activity) after our group manipulation simply because this test was already attached to our battery of problem-solving tasks in our online platform. For sake of continuity, we kept this IQ test where it was. Fortunately, the IQ test’s placement likely did not affect any differences we observed: it was standardized for all participants, IQ scores did not differ between conditions, and IQ is more of a trait measure.

6 As noted, participants in the individual condition had their own performance score for each task. To match the group level of scores for teams, we averaged performance among the four individuals within a given group to construct an average performance score for that group. See supplement ‘Section 4: Alternative scoring methods for groups of individuals’ and Figure S3A/B for details.

7 N ≈ 473 for inter-brain synchrony data, as we had 43 groups x 11 tasks on average where group inter-brain synchrony was computed. N = 308 for group performance data, as we had 44 groups x 7 tasks with performance data.

8 R^2^ values refer to Nakagawa-Schielzeth-Johnson’s (N-S-J) conditional coefficients of determination for generalized mixed-effect models (Nagakawa and Schielzeth, 2013; Johnson, 2014).

9 See supplement Table S1 for zero-order correlations of key variables.
