# Supplementary material for: Inter-brain synchrony in teams predicts collective performance
Source: Soc Cogn Affect Neurosci. 2020 Sep 30;16(1-2):43–57. doi: 10.1093/scan/nsaa135 (PMC7812618; doi:10.1093/scan/nsaa135)
Supplement: nsaa135_Supp [file nsaa135_supp.zip › scan-20-001-File011.docx]

**Supplement**

[Section 1: Methodological Details 2](#_Toc51017590)

[Section 2: Additional items 2](#_Toc51017591)

[Section 3: Task descriptions and scoring 3](#_Toc51017592)

[Section 4: Alternative scoring methods for groups of individuals 4](#_Toc51017593)

[Section 5: Group inter-brain synchrony data loss 5](#_Toc51017594)

[Section 6: Supplemental discussion 6](#_Toc51017595)

[Section 7: References 8](#_Toc51017596)

[Table S1: Zero-order correlations of key variables 11](#_Toc51017597)

[Table S2: Missing channel info 12](#_Toc51017598)

[Table S3: Mixed model predicting collective performance 13](#_Toc51017599)

[Figure S1: Demographics 14](#_Toc51017600)

[Figure S2A/B: EEG recording software 15](#_Toc51017601)

[Figure S3A: Performance (top performer) 16](#_Toc51017602)

[Figure S3B: Performance (pooled) 17](#_Toc51017603)

[Figure S4A/B: Performance correlation matrix 18](#_Toc51017604)

[Figure S5: Counts of group synchrony per task 19](#_Toc51017605)

[Figure S6A: Synchrony in teams vs. individuals (max-min norm) 20](#_Toc51017606)

[Figure S6B: Synchrony in teams vs. individuals over-time (max-min norm) 21](#_Toc51017607)

[Figure S6C: Synchrony in teams vs. individuals at End-baseline (max-min norm) 22](#_Toc51017608)

[Figure S6D: Synchrony in teams vs. individuals (per frequency) 23](#_Toc51017609)

[Figure S6E: Synchrony in teams vs. individuals over-time (per frequency) 24](#_Toc51017610)

[Figure S6F: Synchrony in teams vs. individuals at End-baseline (per frequency) 25](#_Toc51017611)

[Figure S7A: Performance ~ Synchrony (max-min norm) 26](#_Toc51017612)

[Figure S7B: Performance ~ Synchrony (per frequency) 27](#_Toc51017613)

[Figure S7C: Performance ~ Synchrony (per task) 28](#_Toc51017614)

## Section 1: Methodological Details

Although we told participants they could move somewhat naturally in their seat, we asked them to avoid excessive body or head movement. To make this point clear, we showed them how the EEG signal became noisy when they moved their head around, clenched their jaw, wiggled their ears, blinked excessively, etc. using EMOTIV’s TestBench software program that showed them their raw EEG trace in real-time. See Figure S2 Panel A.

## Section 2: Additional items

Cooperation: After making their decision regarding how much (if any) to contribute to the public pot, participants answered four comprehension questions to ensure they understood the task, and then briefly explained, in writing, why they decided to contribute the amount they did. Participants were asked:

1. “What level of contribution earns the highest payoff for the group as a whole?”
   1. response options went from 0 to 1,000 cents (i.e, $10), in increments of 50 cents
2. “What level of contribution earns the highest for you personally?”
   1. response options went from 0 to 1,000 cents (i.e, $10), in increments of 50 cents
3. “What happens to the money that is contributed to the common pot?” Options:
   1. “it gets halved”
   2. “it gets doubled”
   3. “it gets tripled”
   4. “nothing”
4. “Subsequently the money in the common pot gets distributed...” Options:
   1. “evenly distributed among the four players”
   2. “unevenly distributed among the four players”

We found that 10 people answered question #1 incorrectly (correct answer being to give all $10), and 50 people answered question #2 incorrectly (correct answer being to give none). However, the first two comprehension questions can be a bit complicated as payoffs depend on what other group members contribute. When analyzing question #3 and #4, we found that 15 participants answered question #3 incorrectly (correct answer being “it gets doubled”), and 7 answered question #4 incorrectly (correct answer being “evenly distributed among the four players”), with 3 participants answering both #3 and #4 incorrectly. Nevertheless, we did not exclude any participants based on these comprehension questions.

Reading Mind in the Eyes Test: After completing this test, participants answered two questions regarding whether they had taken this “eyes & emotions test” before (85% had not, 7% were unsure, 7% had taken it; we did not exclude any participants) and how capable they felt they were at reading other people’s emotions compared to their family and friends on a 5-point scale, where 1 = much worse, 2 = slightly worse, 3 = about the same, 4 = slightly better, 5 = much better (*M* = 3.6, *SD* = 1.0). Performing a one-sample t-test with the expected mean in the middle of the scale, we find a “better than average” effect, whereby participants consistently believed they were slightly better than their family and friends at reading other people’s emotions, *t*(173) = 8.25, *p* < .001.

## Section 3: Task descriptions and scoring

We selected tasks to range across the McGrath Task Circumplex (Straus, 1989) in terms of skills required to complete them. *Generating* tasks required the generation of new information and ideas. *Choosing* tasks involve deciding about issues that either have a correct answer or which are matters of judgment; intellective and judgmental tasks, respectively. *Negotiation* tasks involve reasoning through conflicts of interest or points of view. *Executing* tasks involve physical performances and psycho-motor tasks. *Remembering* tasks required participants to collectively store and retrieve information, similar to those evaluated in work on transactive memory systems.

**IQ test** (Choosing task)**:** Participants were given 6 minutes to individually complete a 20-question subset of the Raven’s Advanced Progressive Matrices test. All of the questions on the Raven's progressives consist of visual geometric design with a missing piece. The test taker is given six to eight choices to pick from and fill in the missing piece. Answers were either correct or incorrect. Scores represent the proportion of questions answered correctly (x/20).

**Photo/Memory** (Remembering task)**:** Individuals/teams were given 90 seconds to study a busy image. Then the photo disappeared, and individuals/teams were given 2 minutes to answer 6 questions about the photo from memory. Answers were either correct or incorrect. Scores represent the proportion of questions answered correctly (x/6).

**Typing** (Execution task)**:** Individuals/teams had 6.5 minutes to re-type a passage of text as accurately as possible. Typed passages of text were cut to the last word of the user submission and then compared with the original passage of text via: <http://countwordsfree.com/comparetexts>. Levenshtein’s distance^^[[1]](#footnote-1)^^, a string metric for measuring the difference between two sequences, was computed, with higher numbers indicating more errors in typing. We then standardized each Levenshtein’s score by the number of characters typed (i.e., Levenshtein’s distance / character count). Standardizing ensured that someone who mis-typed one word but only wrote ten words would be penalized more than someone who also mis-typed one word but wrote a full page of text. Finally, we took the multiplicative inverse of the standardized Levenshtein’s distance score to arrive at a “correctness” score, with higher scores indicating more accurate typing of text (i.e., 1 / (Levenshtein’s distance / character count)).

**Brainstorm** (Generation task)**:** Individuals/teams had 2 minutes to generate creative uses of a brick. Each idea generated by participants/teams was automatically compared against an existing database of different creative “use” categories (e.g., “break a window” is a different “use” category than “weigh something down”). Each idea that tagged a “use” category was given one point. If an idea was unique among our study’s participants, it could also gain up to one additional point (a “uniqueness” bonus). Specifically, the uniqueness bonus was calculated as 1 - proportion of other groups who had also mentioned that specific “use” category. For example, if every group answered one example from the category “build a wall”, the score for that would be 1 point (i.e., not unique at all, thus no uniqueness bonus). However, if only half of the groups answered from the category “use as paperweight”, the score for that would be 1.5 points (i.e., somewhat unique, thus 0.5 uniqueness bonus added to the base 1 point for the idea).

**Unscramble** (Choosing task)**:** Individuals/teams had 4 minutes to unscramble 24 words. Answers were either correct or incorrect. Scores represent the proportion of questions answered correctly (x/24).

**Sudoku** (Choosing task)**:** Individuals/teams had 3.5 minutes to complete a partially filled in Sudoku puzzle (there were 47 blank squares needing to be filled in). Answers were either correct or incorrect. Scores represent the proportion of squares correctly filled in (x/47).

**Winter Survival** (Negotiation task)**:** Individuals/teams had 10 minutes to (1) read a short scenario describing a plane crash in the middle of the winter in the woods in northern Minnesota, and (2) rank order 15 objects remaining from the plane crash in terms of the object’s importance for their (or their team’s) survival. Every object had an “objective” rank spot (pre-determined by survival experts). As per the scoring instructions for this task, we calculated difference scores from participant’s/team’s answers and the correct rank spot and summed all the difference scores. Thus, larger numbers indicated larger discrepancies between a participant’s/team’s ranking and the correct ranking (i.e., less accurate rankings). To flip scores such that larger numbers indicated more accurate rankings, we then inverted the scores by taking the maximum possible total sum of difference scores (112) and subtracting a participant’s/team’s score.

## Section 4: Alternative scoring methods for groups of individuals

While teams worked together and received a single score for each task, participants in the individual condition worked independently and thus each have their own score within their group of four competitors. As noted in the main text, we averaged performance among the four individuals within a given group to construct an average performance score for that group for each task. Thus, we compared teams against an “average individual”. However, some research suggests that “two heads are better than one” (Hill, 1982), implying that a group can outperform even the best member of a group, and experience a “collective benefit” (Bahrami et al., 2010; Kerr & Tindale, 2004; Wahn et al., 2018; Woolley et al., 2010). Thus, instead of averaging across individuals, we can simply select the best individual within a group for each task and compare that top-individual performer against our teams (note that the top-performer may not always be the same individual across all tasks; rather it is simply the individual within a group of four competitors who did the best on a given task). As seen in Figure S3A, teams still outperformed the top-individual performer across several tasks, although no longer outperformed on the memory task, and did worse on the winter survival task (and as before, continued to do worse on the typing task).

Moreover, as a strict test of whether groups provide “collective benefit” beyond what an equal number of individuals could achieve separately, we also examined the three tasks where it was possible to “pool” individual’s responses to maximize their performance. In other words, if one individual within a group (in the individual condition) got a specific answer to a question correct, but no other competitor in their group got that answer correct, the “group” would still get that question correct. In a way, this assumes that had these four individuals worked together, the one individual who knew the right answer to that question would have been able to convince the other group members that theirs was indeed the correct response. As seen in Figure S3B, teams did no worse that the optimally pooled group of individuals on unscrambling words and sudoku. However, teams did worse on the memory task, suggesting a “collective forgetting” effect, as teams used the chat window to converse and recall aspects of the image, possibly eliciting a socially based retrieval-induced forgetting effect (Coman et al., 2009; Stone & Hirst, 2014).

## Section 5: Group inter-brain synchrony data loss

Given that each group had 14 tasks where EEG was recorded and that we had 44 groups, no data loss would have meant 616 tasks worth of EEG data. However, after pre-processing we were left with only 474 tasks worth of EEG data (23% data loss), including the removal of one group’s EEG data altogether. This level of data loss is in keeping with previous research utilizing EMOTIV EPOC wireless EEG headsets (see Dikker et al., 2017).

Of the 474 remaining tasks and of 14 possible channels from the EMOTIV EPOC wireless headset, most channels from most subjects were available for synchrony computations for a given task (see Table S2). Table S2 displays the % missing for each possible number of missing channel(s) across all subjects and all tasks. Note however, that while these numbers reflect all 14 possible channels, we only averaged coherence values across 1-20 Hz over 6 electrode pairs of interest ('F3' 'F4' 'O1' 'O2' 'P7' 'P8') to stay consistent with the inter-brain synchrony approach used in Dikker et al. (2017) and Bevilacqua et al., (2019). As noted in our main text, these 6 electrodes were most likely to yield decent data quality (see Figure 1C; Dikker et al., 2017), and focusing on these channels may help mitigate discrepancies between groups regarding the number of channels that were considered for analysis (due to differences in channel removal during pre-processing).

Moreover, the majority of our group inter-brain synchrony data indeed reflects group synchrony between all members of the group. Of the remaining 474 tasks: 85% had all six possible pairwise synchrony scores available (with four participants there are six possible unique pairwise combinations), 1.5% had five pairwise synchrony scores, 11.2% had only three pairwise synchrony scores, and 2.3% had only one pairwise synchrony score.

Finally, as noted in the main text section “EEG preprocessing”, our preprocessing pipeline resulted in the preservation of an average of 11 of 14 tasks per group in both the individual and team condition. Importantly, when examining the number of group synchrony values, there was no significant difference between the team and individual condition across tasks, *t*(18.94) = 0.82, *p* = .421 (see Figure S5).

## Section 6: Supplemental discussion

Much of the existing literature suggests that inter-brain synchrony is associated with neural signatures commonly associated with attention (e.g., synchronization in the alpha band; Dumas et al., 2010; Lachat et al., 2012; Hasegawa et al., 2016; Szymanski et al., 2017), motor planning (e.g., mu/beta synchronization; Dumas et al., 2010; Konvalinka et al., 2014; Mu et al., 2016), or mentalizing (e.g., synchronization via TPJ; Bilek et al., 2015; Jahng et al., 2017; Jiang et al., 2015; Tang et al., 2016; Zhang et al., 2017) between face-to-face partners. One common interpretation of inter-brain synchrony is that it may reflect shared attention to a common stimulus. During social interaction, however, such shared entrainment may be ‘overshadowed’ by social coordination demands, particularly to an in-person social partner. This was shown nicely in recent work which disentangled inter-brain synchrony effects due to shared stimulus entrainment from inter-brain synchrony associated with conversational alignment in dyads (Perez et al., 2017).

In the present study, the stimuli were arguably equally engaging (entraining) between groups of individuals and teams, but the number of events in the team vs. individual condition may have impacted the level of entrainment in some teams (i.e., perhaps uncoordinated social demands overshadowed stimulus entrainment). For example, while individual participants were invited to use the chat box for note-taking, this occurred much less frequently than in the team condition (only 24 participants in the individual condition did so versus all 87 participants who were a part of teams), resulting in more stretches in the data where all participants in the individual condition were focusing on the same sensory stimulus instead of engaging in actions that might not be time-locked to each other (e.g., typing or reading the chat). In fact, ineffective teams may suffer from group issues such as social loafing (Latané et al., 1979), and this lack of effort and engagement might be predicted by the extent to which attentional distractions occur, resulting in a reduction in inter-brain synchrony and worse team performance. Of course, other work has shown that interactive social engagement, which is associated with more instances of social coordination, in fact *increases* inter-brain synchrony (e.g., Pan et al., 2019). However, in our study participants were interacting by typing, which is not as temporally coordinated as conversational interactions altering the interactive dynamics (McDaniel et al., 1996). As such, it is possible that entrainment to a common stimulus – the computer tasks – creates a basis for inter-brain synchrony, which can then be moderated by social context and consequently track socially relevant variables.

It is also worth noting that we did not fully remove the social interactive aspect from our individual condition: four participants were still in the same room, sitting next to/across from each other at the same table, and well aware of the competitive aspect of doing well on the problem solving tasks individually (e.g., hearing the rapid typing of text from a competitive participant often noticeably amped up the competitive spirit in the room among competing individuals). Indeed, while much of the literature has found inter-brain synchrony to be related to cooperative periods, some research has also found inter-brain synchrony during competitive periods, which can also require a form of attention and mentalizing to strategize against an opponent (Liu et al., 2015; Liu et al., 2017). While past research has sought to tease apart the distinction between shared sensory input and social interaction on dyadic team performance -- finding greater inter-brain synchrony in a visual search task done jointly versus independently and that inter-brain synchrony tracked performance gains (Szymanski et al., 2017) -- that work had removed more of the social element (participants were seated at separate tables and faced away from each other, and there was no auditory component at all). Nevertheless, as we found that inter-brain synchrony tracks performance among teams, we conceptually replicate and extend the work from Szymanski et al (2017).

Although past research has suggested that sharing a group identity can help improve collective achievements (Hogg et al., 2004; Hertel & Solansky, 2011; Baumeister et al., 2016), we did not find that self-reported group identification predicted collective performance. Indeed, group identification may not be related to collective performance for groups who are only working together for a brief period of time (compared to groups who repeatedly work together).

Additional Limitations

There are various ways to compute inter-brain synchrony, with no agreed upon single “best” approach. In fact, different synchrony metrics may predict different cognitively relevant processes (Dikker, Michalareas et al., 2019). Here, we chose our metric of interest (coherence) to be consistent with the purposes of our previous group synchrony research, where we found that inter-brain coherence predicted various social and stimulus-related factors using the same low-density EEG headsets (Dikker et al., 2017; Bevilacqua et al., 2019). Future research may benefit from using a “multiverse” strategy (Steegan et al., 2016) to explore how various decisions to compute synchrony affect the results.

In addition, due to technical issues, video recordings of the ongoing interactions were not perfectly synchronized with the EEG data, which limited our ability to use the videos to identify specific behaviors associated with increases or decreases in inter-brain synchrony. Some inter-brain synchrony research has done this to a fruitful end (e.g., Kinreich et al., 2017), but future research should continue to integrate video recordings of social interactions to assess the behaviors that correspond to inter-brain synchrony.

## Section 7: References

Bahrami, B., Olsen, K., Latham, P. E., Roepstorff, A., Rees, G., & Frith, C. D. (2010). Optimally interacting minds. *Science*, *329*(5995), 1081-1085.

Baumeister, R. F., Ainsworth, S. E., & Vohs, K. D. (2016). Are groups more or less than the sum of their members? The moderating role of individual identification. Behavioral and Brain Sciences, 39.

Bevilacqua, D., Davidesco, I., Wan, L., Chaloner, K., Rowland, J., Ding, M., ... & Dikker, S. (2019). Brain-to-brain synchrony and learning outcomes vary by student–teacher dynamics: Evidence from a real-world classroom electroencephalography study. Journal of cognitive neuroscience, 31(3), 401-411.

Bilek, E., Ruf, M., Schäfer, A., Akdeniz, C., Calhoun, V. D., Schmahl, C., ... & Meyer-Lindenberg, A. (2015). Information flow between interacting human brains: Identification, validation, and relationship to social expertise. Proceedings of the National Academy of Sciences, 112(16), 5207-5212.

Coman, A., Manier, D., & Hirst, W. (2009). Forgetting the unforgettable through conversation: Socially shared retrieval-induced forgetting of September 11 memories. Psychological Science, 20(5), 627-633.

Dikker, S., Michalareas, G., Oostrik M., Serafimaki, A., Kahraman, H.A., Struiksma, M. E., Poeppel, D. (2019). Crowdsourcing neuroscience: inter-brain coupling during face-to-face interactions outside the laboratory. biorxiv: https://www.biorxiv.org/content/10.1101/822320v1

Dikker, S., Wan, L., Davidesco, I., Kaggen, L., Oostrik, M., McClintock, J., ... & Poeppel, D. (2017). Brain-to-brain synchrony tracks real-world dynamic group interactions in the classroom. Current Biology, 27(9), 1375-1380.

Dumas, G., Nadel, J., Soussignan, R., Martinerie, J., & Garnero, L. (2010). Inter-brain synchronization during social interaction. PloS one, 5(8), e12166.

Hasegawa, C., Ikeda, T., Yoshimura, Y., Hiraishi, H., Takahashi, T., Furutani, N., ... & Kikuchi, M. (2016). Mu rhythm suppression reflects mother-child face-to-face interactions: a pilot study with simultaneous MEG recording. Scientific reports, 6, 34977.

Hertel, G., & Solansky, S. T. (2011). Team identification: a determining factor of performance. Journal of Managerial Psychology, 26(3), 247-258.

Hogg, M. A., Abrams, D., Otten, S., & Hinkle, S. (2004). The social identity perspective: Intergroup relations, self-conception, and small groups. Small group research, 35(3), 246-276.

Jahng, J., Kralik, J. D., Hwang, D. U., & Jeong, J. (2017). Neural dynamics of two players when using nonverbal cues to gauge intentions to cooperate during the Prisoner's Dilemma Game. Neuroimage, 157, 263-274.

Jiang, J., Chen, C., Dai, B., Shi, G., Ding, G., Liu, L., & Lu, C. (2015). Leader emergence through interpersonal neural synchronization. Proceedings of the National Academy of Sciences, 112(14), 4274-4279.

Kerr, N. L., & Tindale, R. S. (2004). Group performance and decision making. Annu. Rev. Psychol., 55, 623-655.

Kinreich, S., Djalovski, A., Kraus, L., Louzoun, Y., & Feldman, R. (2017). Brain-to-brain synchrony during naturalistic social interactions. Scientific reports, 7(1), 17060.

Konvalinka, I., Bauer, M., Stahlhut, C., Hansen, L. K., Roepstorff, A., & Frith, C. D. (2014). Frontal alpha oscillations distinguish leaders from followers: multivariate decoding of mutually interacting brains. Neuroimage, 94, 79-88.

Lachat, F., & George, N. (2012). Oscillatory brain correlates of live joint attention: a dual-EEG study. Frontiers in human neuroscience, 6, 156.

Latané, B., Williams, K., & Harkins, S. (1979). Many hands make light the work: The causes and consequences of social loafing. Journal of personality and social psychology, 37(6), 822.

Liu, T., Saito, G., Lin, C., & Saito, H. (2017). Inter-brain network underlying turn-based cooperation and competition: A hyperscanning study using near-infrared spectroscopy. Scientific reports, 7(1), 8684.

Liu, T., Saito, H., & Oi, M. (2015). Role of the right inferior frontal gyrus in turn-based cooperation and competition: a near-infrared spectroscopy study. Brain and cognition, 99, 17-23.

McDaniel, S. E., Olson, G. M., & Magee, J. C. (1996, November). Identifying and analyzing multiple threads in computer-mediated and face-to-face conversations. In Proceedings of the 1996 ACM conference on Computer supported cooperative work (pp. 39-47). ACM.

Mu, Y., Guo, C., & Han, S. (2016). Oxytocin enhances inter-brain synchrony during social coordination in male adults. Social cognitive and affective neuroscience, 11(12), 1882-1893.

Pan, Y., Dikker, S., Goldstein, P., Zhu, Y., Yang, C., Hu, Y. (2019). Instructor-learner brain coupling discriminates between instructional approaches and predicts learning. BioRxiv: <https://www.biorxiv.org/content/10.1101/704239v2>

Pérez, A., Carreiras, M., & Duñabeitia, J. A. (2017). Brain-to-brain entrainment: EEG interbrain synchronization while speaking and listening. Scientific reports, 7(1), 4190

Steegen, S., Tuerlinckx, F., Gelman, A., & Vanpaemel, W. (2016). Increasing transparency through a multiverse analysis. Perspectives on Psychological Science, 11(5), 702-712.

Stone, C. B., & Hirst, W. (2014). (Induced) Forgetting to form a collective memory. Memory Studies, 7(3), 314-327.

Szymanski, C., Pesquita, A., Brennan, A. A., Perdikis, D., Enns, J. T., Brick, T. R., ... & Lindenberger, U. (2017). Teams on the same wavelength perform better: Inter-brain phase synchronization constitutes a neural substrate for social facilitation. Neuroimage, 152, 425-436.

Tang, H., Mai, X., Wang, S., Zhu, C., Krueger, F., & Liu, C. (2015). Interpersonal brain synchronization in the right temporo-parietal junction during face-to-face economic exchange. Social cognitive and affective neuroscience, 11(1), 23-32.

Wahn, B., Czeszumski, A., & König, P. (2018). Performance similarities predict collective benefits in dyadic and triadic joint visual search. *PloS one*, *13*(1), e0191179.

Woolley, A. W., Aggarwal, I., & Malone, T. W. (2015). Collective intelligence and group performance. Current Directions in Psychological Science, 24(6), 420-424.

Zhang, M., Liu, T., Pelowski, M., Jia, H., & Yu, D. (2017). Social risky decision-making reveals gender differences in the TPJ: a hyperscanning study using functional near-infrared spectroscopy. Brain and cognition, 119, 54-63.

## Table S1: Zero-order correlations of key variables

|  |  | 1 | 2 | 3 | 4 | 5 | 6 |
| --- | --- | --- | --- | --- | --- | --- | --- |
| 1 | Cooperation | -- | .18* | .03 | .08 | .16* | .33^*^ |
| 2 | Group Identification |  | -- | .22** | .07 | .28* | .00 |
| 3 | Groupiness |  |  | -- | -.06 | .08 | .01 |
| 4 | Emotion Perception |  |  |  | -- | .23** | .11 |
| 5 | Collective Performance |  |  |  |  | -- | .07 |
| 6 | Inter-brain synchrony |  |  |  |  |  | -- |

Zero-order correlations key variables collapsed across conditions. Notes: ^†^p < .10, **p* < .05, ***p* < .01. All variables are at the subject level except performance and inter-brain synchrony, which are at the group level (and thus any correlations with those two variables are also at the group level).

## Table S2: Missing channel info

| # of possible missing channels | % missing across subjects and tasks |
| --- | --- |
| 1 | 36.0% |
| 2 | 27.4% |
| 3 | 12.3% |
| 4 | 7.7% |
| 5 | 5.5% |
| 6 | 3.1% |
| 7 | 1.4% |
| 8 | 0.6% |
| 9 | 0.7% |
| 10 | 0.06% |
| 11 | -- |
| 12 | -- |
| 13 | -- |
| 14 | 5.3% |

## Table S3: Mixed model predicting collective performance

| **Variable** | **Step 1** | **Step 2** | **Step 3** |
| --- | --- | --- | --- |
| Intercept | -0.23 (0.08)** | -0.20 (0.10)^*^ | -0.20 (0.10)^*^ |
| Team (vs. Individual) | 0.54 (0.11)** | 0.46 (0.14)** | 0.47 (0.14)** |
| Inter-brain synchrony | -0.15 (0.08)^†^ | -0.15 (0.08)^†^ | -0.15 (0.08)^†^ |
| Inter-brain synchrony*Team | 0.35 (0.11)** | 0.35 (0.11)** | 0.35 (0.11)** |
| Group identification |  | 0.00 (0.01) | 0.00 (0.01) |
| Group identification*Team |  | 0.00 (0.01) | 0.00 (0.01) |
| Cooperation |  |  | 0.00 (0.06) |
| Cooperation*Team |  |  | -0.01 (0.09) |
| Emotion perception |  |  | 0.00 (0.03) |
| Emotion perception*Team |  |  | 0.03 (0.07) |
| N-S-J pseudo R^2^ | 14.5% | 14.7% | 14.6% |

Notes: ^†^p < .10, *p < .05, **p < .01. Model results report unstandardized coefficients with standard error in parentheses. All continuous predictors were mean-centered. Inter-brain synchrony was also scaled, taking the centered values and dividing them by its standard deviation. Each step of the model estimated a random intercept nested by group. R^2^ values refer to Nakagawa-Schielzeth-Johnson’s (N-S-J) conditional coefficients of determination for generalized mixed-effect models (see (Johnson, 2014; Nagakawa & Schielzeth, 2013)).

## Figure S1: Demographics


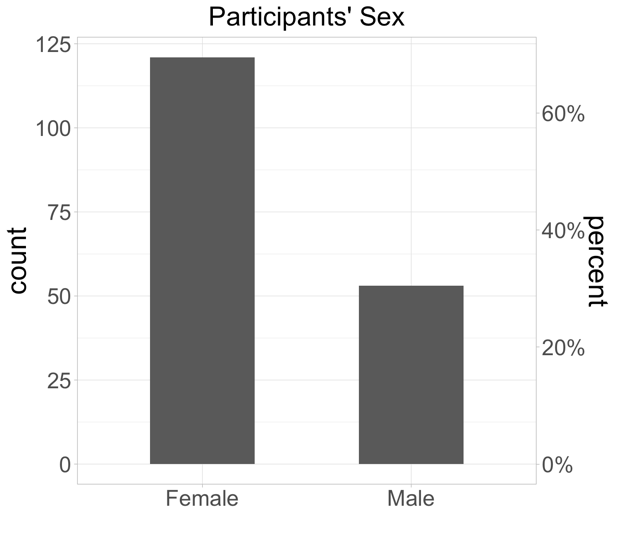

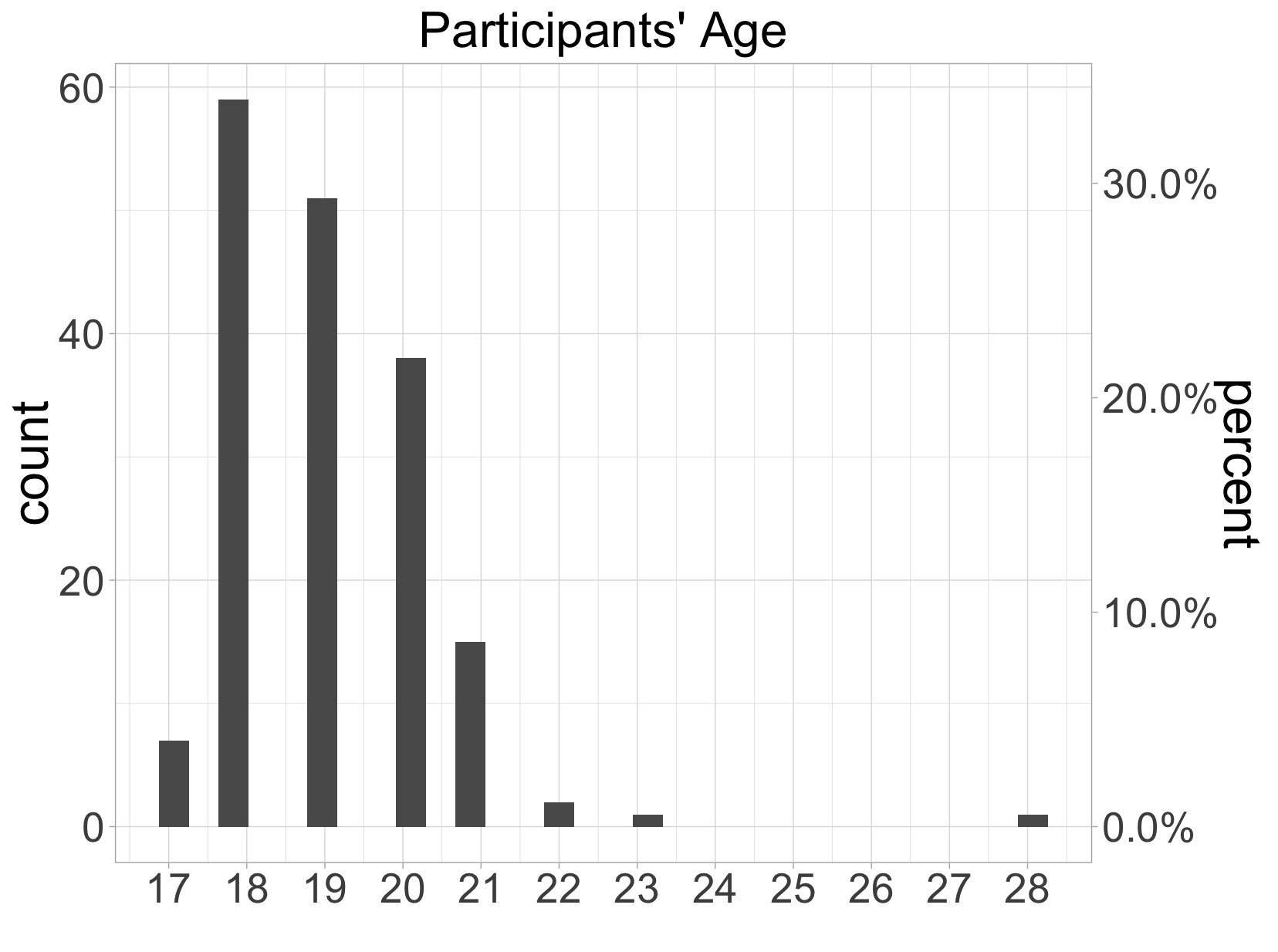


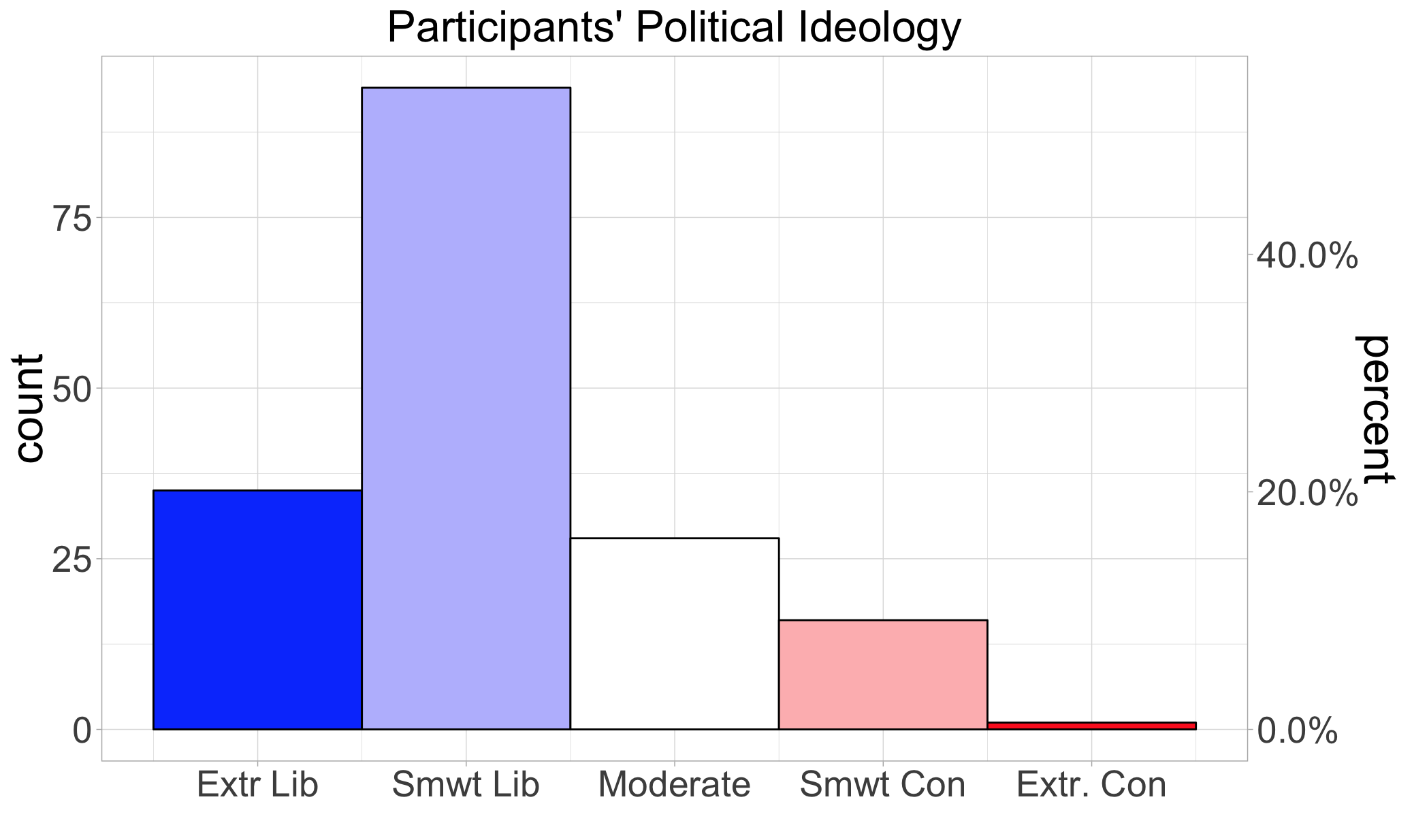

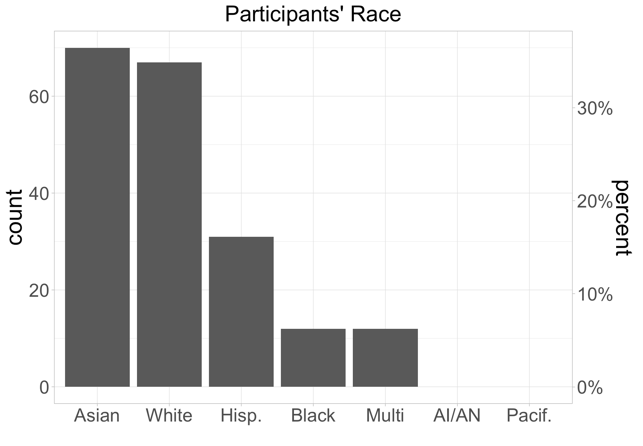


## Figure S2A/B: EEG recording software


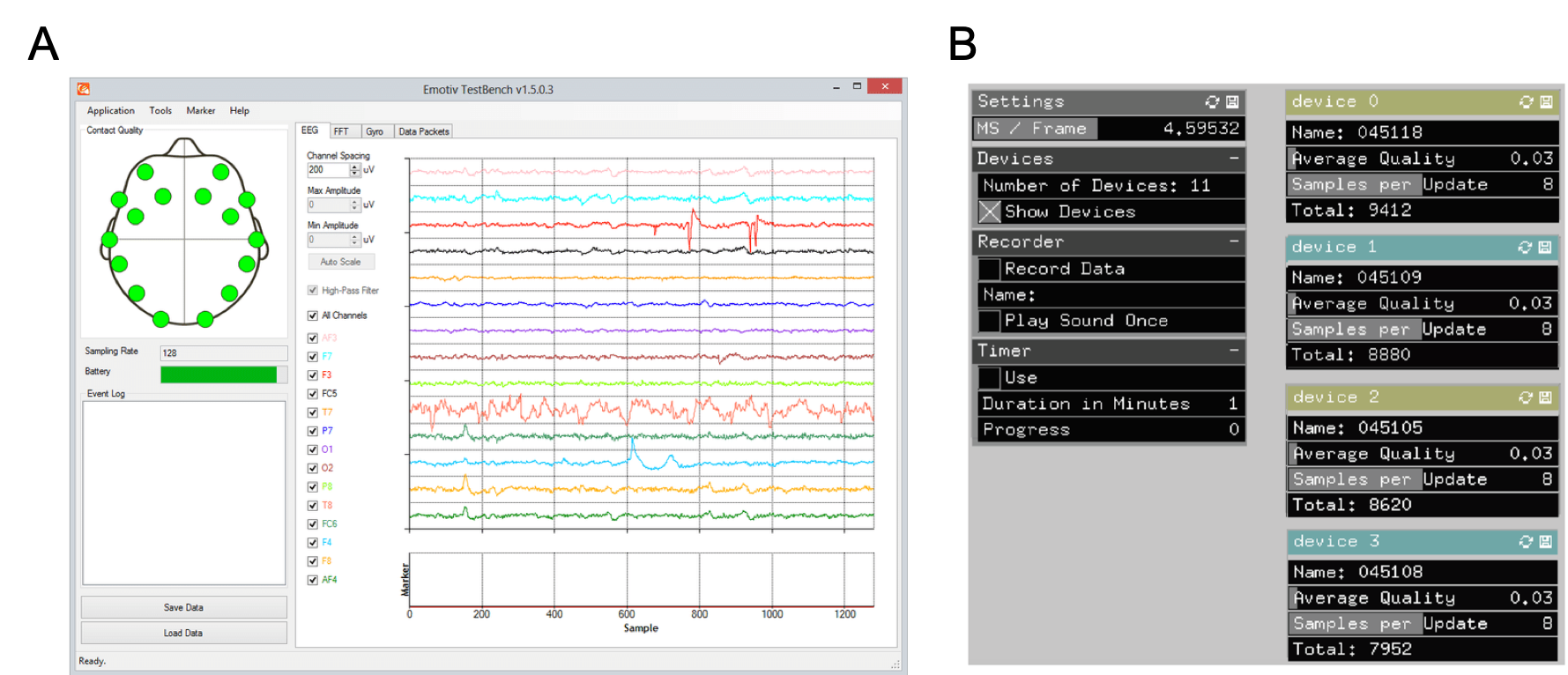


Panel A: screenshot of EMOTIV’s TestBench software program. Panel B: screenshot of the recording software developed by Matthias Oostrik, enabling us to record from multiple EEG headsets simultaneously.

## Figure S3A: Performance (top performer)


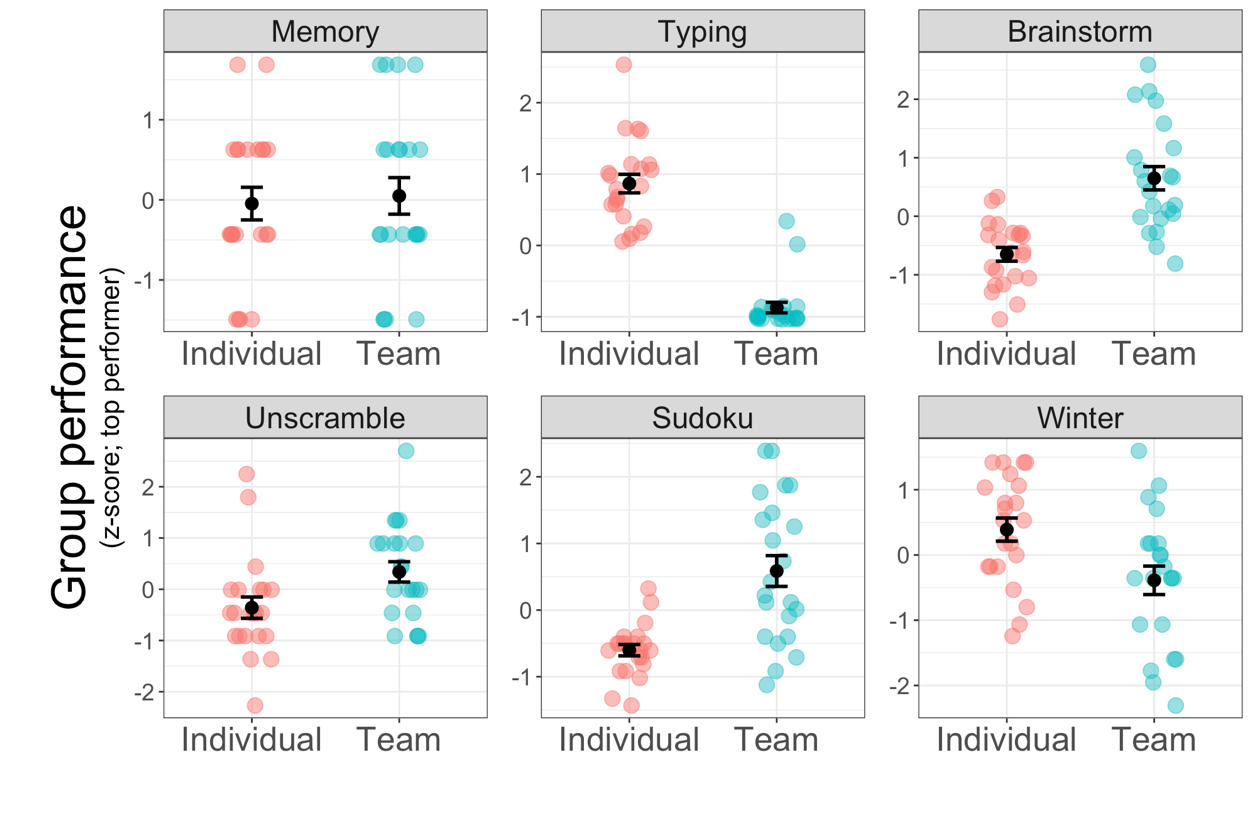


Collective performance of teams (turquoise) and the top-individual performer (red) across all 6 problem-solving tasks. Teams still out-performed the top-individual performer across several tasks, although no longer outperformed on the memory task, and did worse on the winter survival task (and as before, continued to do worse on the typing task). Means are indicated by a black dot with error bars representing +/- 1 standard error.

## Figure S3B: Performance (pooled)


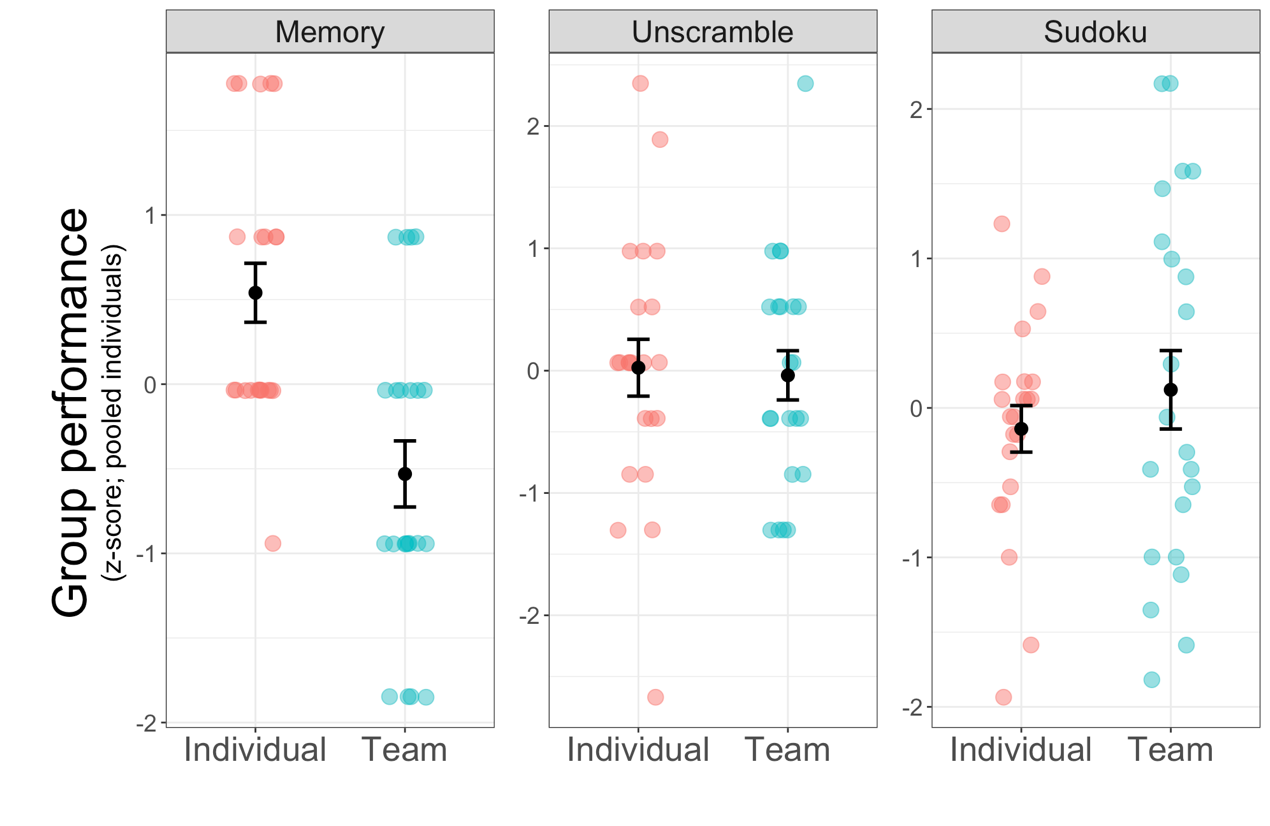


Teams (turquoise) did no worse than optimally pooled groups of individuals (red) on unscrambling words and sudoku, although teams did worse on the memory task. To “pool” individual scores we scored the data such that so long as one competitor within a given group answered a question correctly, the entire group was deemed to have answered that question correctly. Pooling was possible for the 3 tasks above. Means are indicated by a black dot with error bars representing +/- 1 standard error.

## Figure S4A/B: Performance correlation matrix


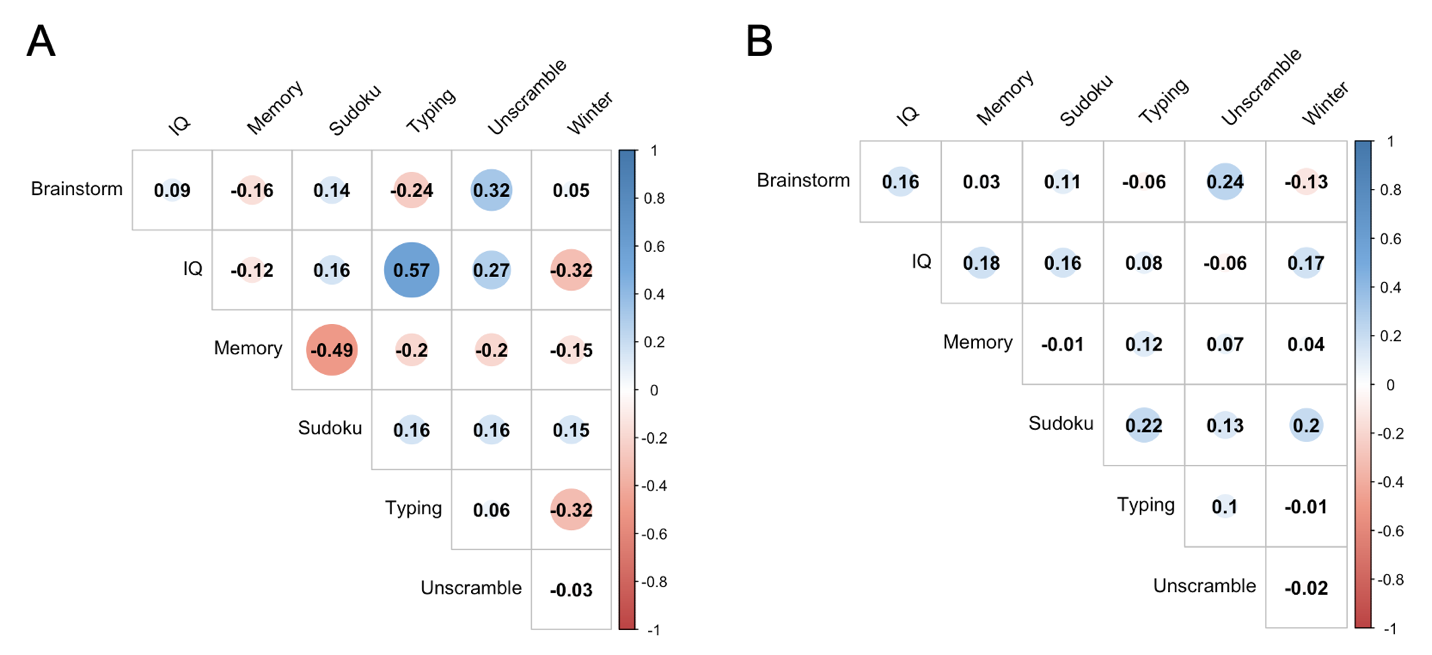


Panel A: Correlation matrix of team performance (z-scored) across tasks. Panel B: Correlation matrix of performance (z-scored) for participants in the individual condition across tasks.

## Figure S5: Counts of group synchrony per task


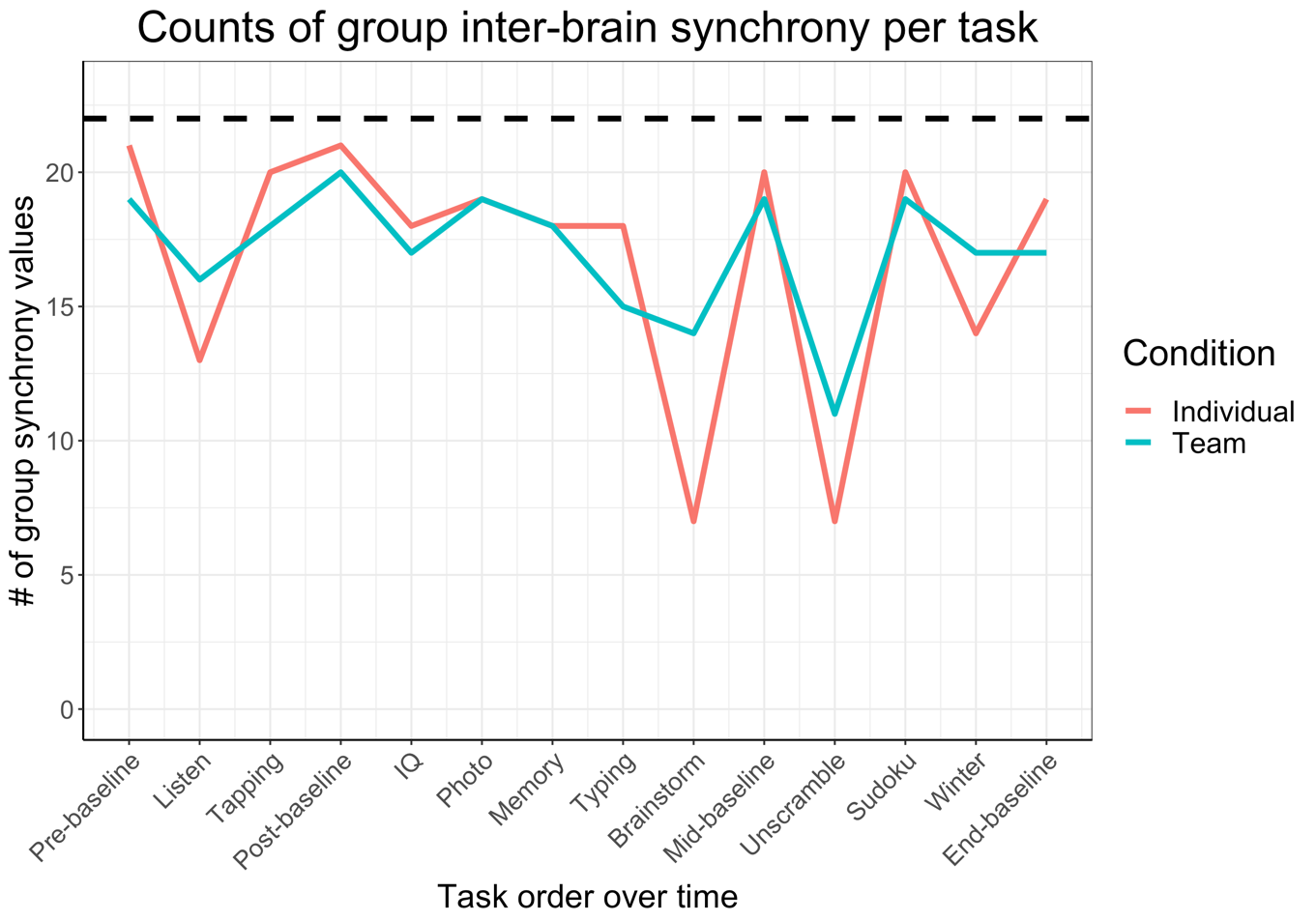


While there was some data loss of group synchrony for each task, data loss was similar across tasks and conditions. The black dashed horizontal line at the indicates what the expected number of group inter-brain synchrony values would have been with no data loss. The two exceptions were brainstorming uses of a brick (individual condition) and unscrambling words (both conditions), which appear to have more data loss relative to the other tasks. Importantly however, there is no significant difference in data loss between the team and individual condition across tasks, *t*(18.94) = 0.82, *p* = .421.

## Figure S6A: Synchrony in teams vs. individuals (max-min norm)


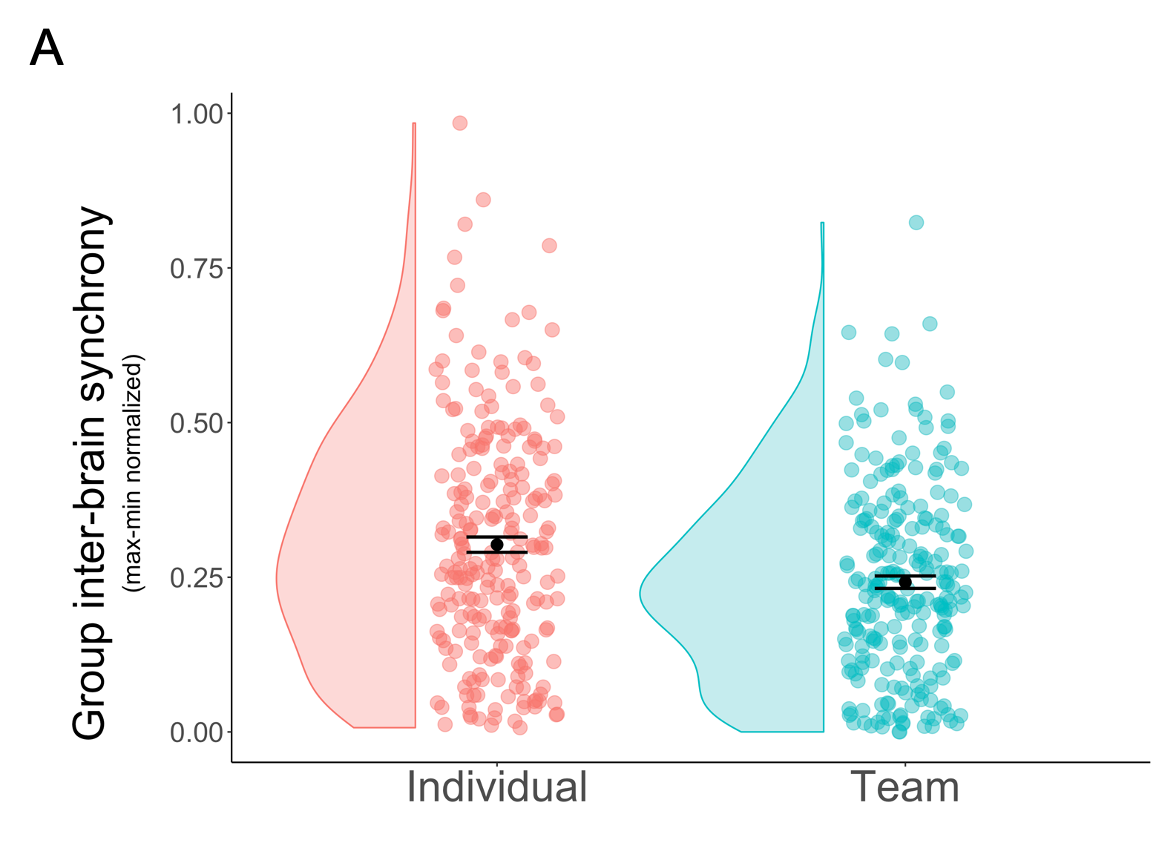


Using max-min normalized (0-1) synchrony values and including all tasks with available EEG data, groups of individuals (red) showed more inter-brain synchrony than teams (turquoise). However, as seen in Figure S6B, this difference was primarily driven by differences at pre-baseline. Each dot represents a group inter-brain synchrony value for a given task. Means are indicated by a black dot with error bars representing +/- 1 standard error.

## Figure S6B: Synchrony in teams vs. individuals over-time (max-min norm)


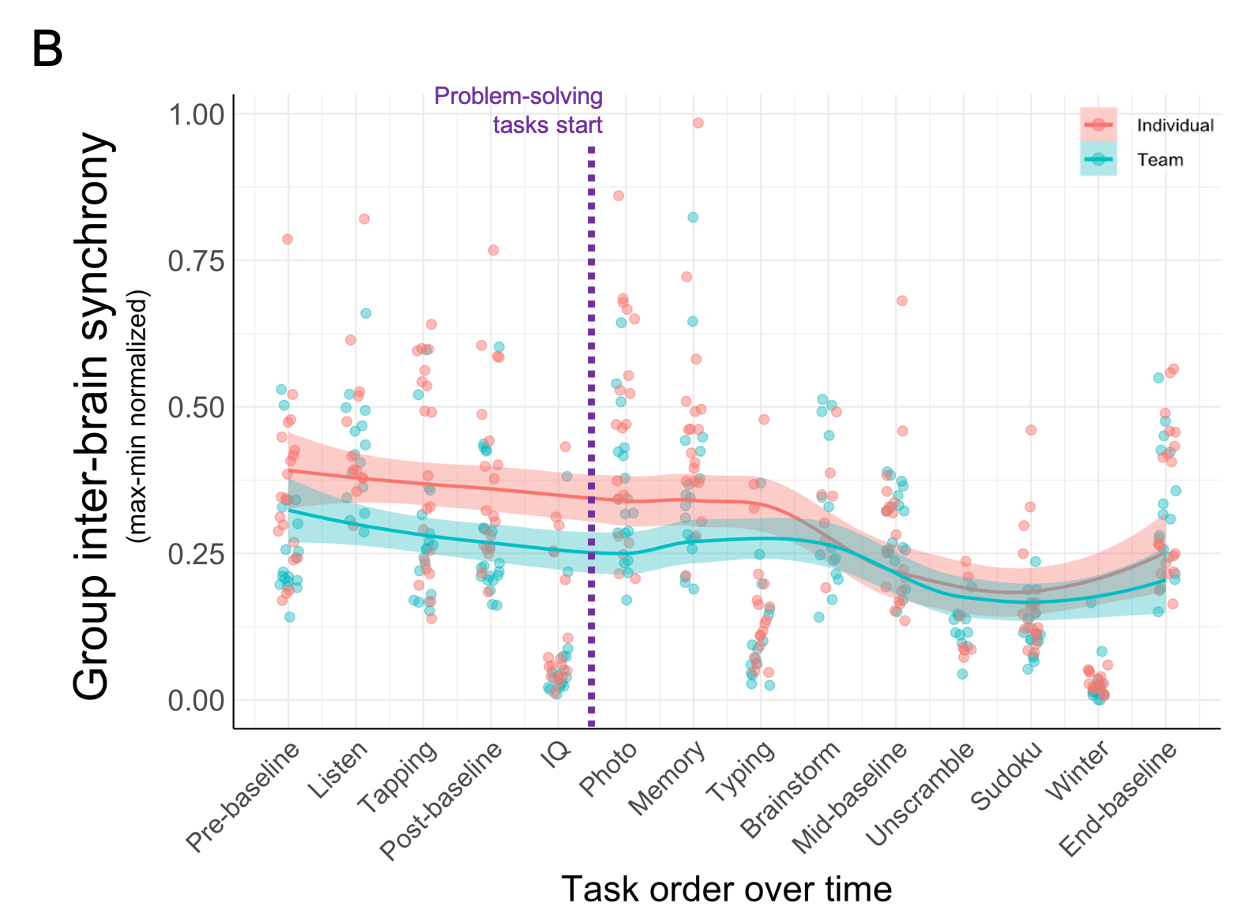


Group inter-brain synchrony decreased over time for both teams (turquoise) and individuals (red), although this decrease was buffered for teams once they began the collective problem-solving tasks. All synchrony values are max-min normalized (0-1). Each dot represents a group inter-brain synchrony value for a given task.

## Figure S6C: Synchrony in teams vs. individuals at End-baseline (max-min norm)


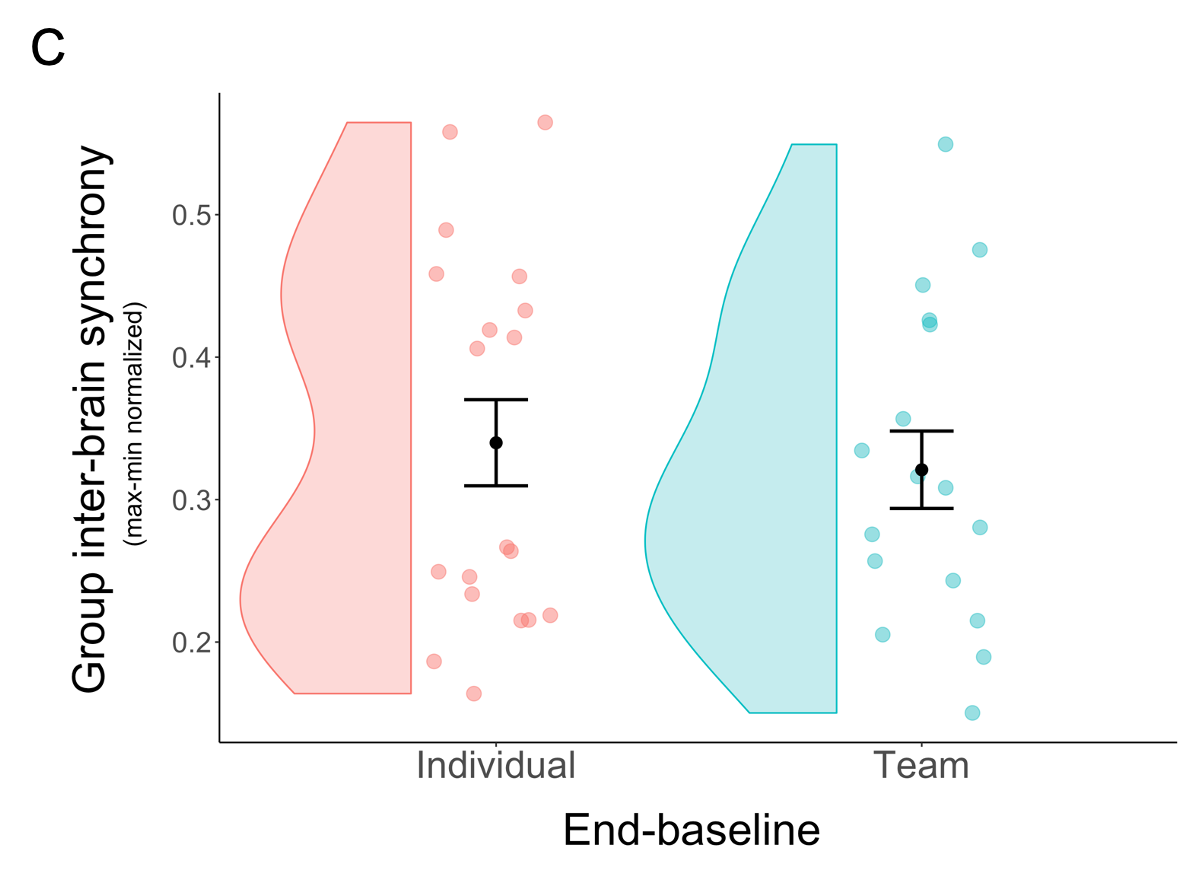


Using max-min (0-1) normalized synchrony values, we see that although groups of individuals (red) had more synchrony at the beginning of the experiment, teams (turquoise) caught up by the end of the experiment (see Figure S6B). Each dot represents a group inter-brain synchrony value during the end-baseline task.

## Figure S6D: Synchrony in teams vs. individuals (per frequency)


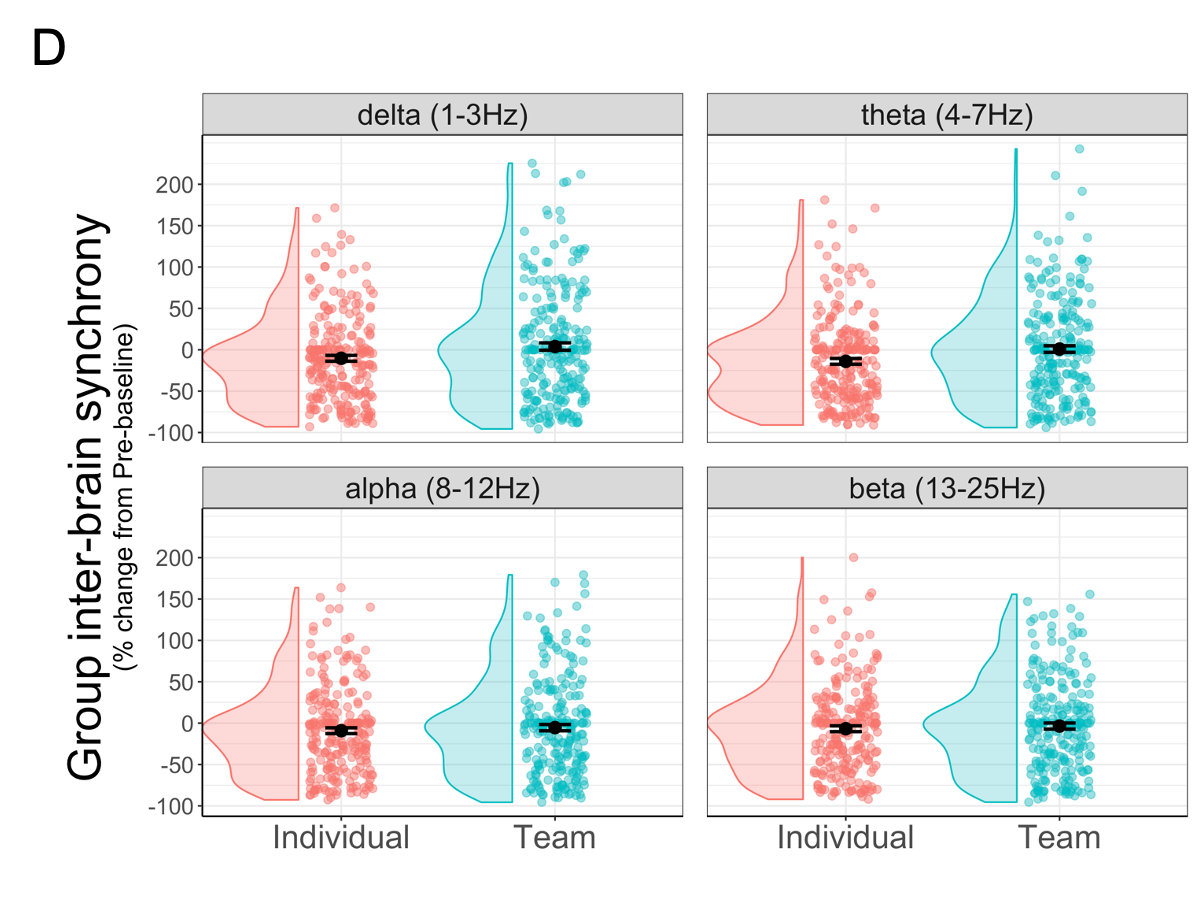


Group inter-brain synchrony was similar between teams (turquoise) and individuals (red) including all tasks with available EEG data and across all frequency bands. All values represent a group’s percent coherence change from pre-baseline, with each dot representing a group inter-brain synchrony value for a given task. Means are indicated by a black dot with error bars representing +/- 1 standard error.

## Figure S6E: Synchrony in teams vs. individuals over-time (per frequency)


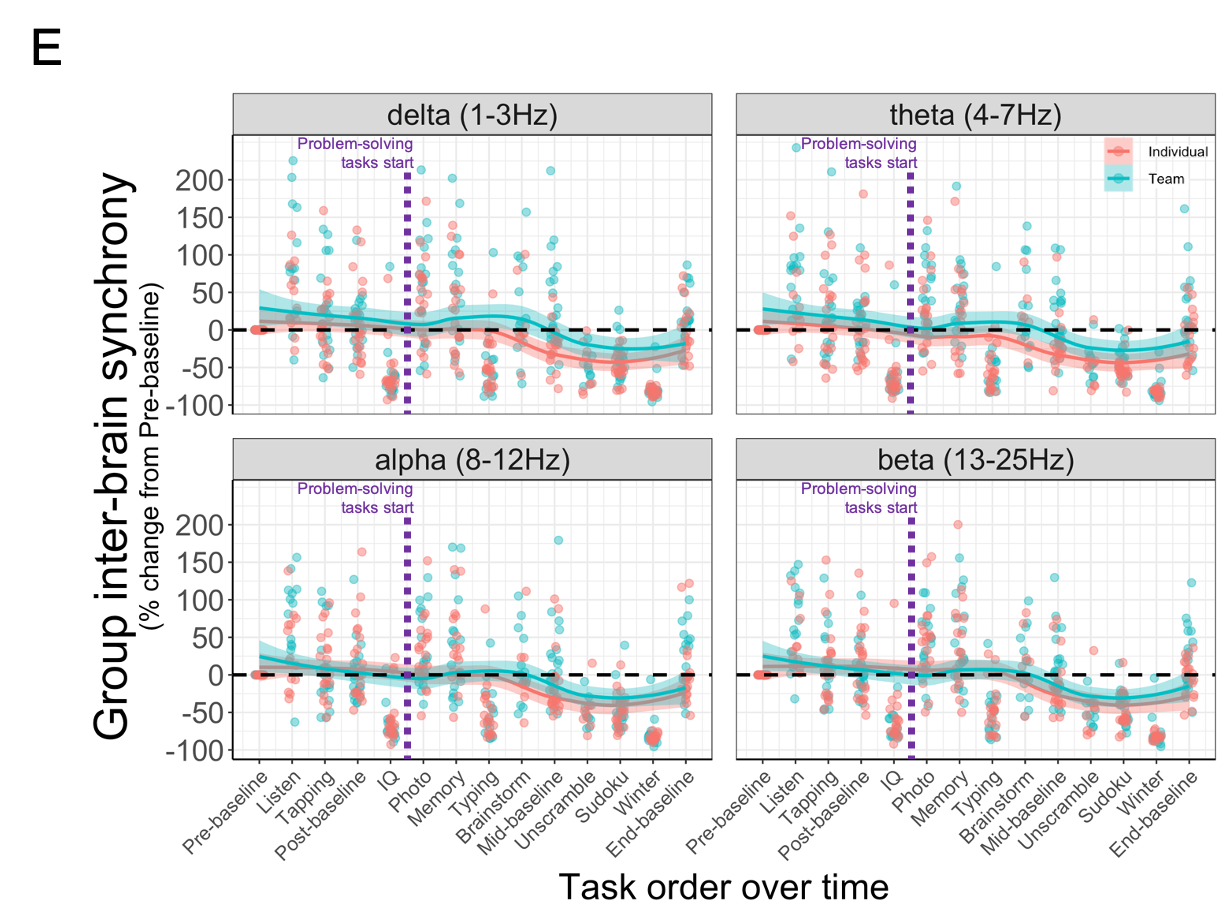


Group inter-brain synchrony decreased over time for both teams (turquoise) and individuals (red) across all frequency bands, although this decrease was slightly buffered for teams once they began the collective problem-solving tasks. All values represent a group’s percent coherence change from pre-baseline, with each dot representing a group inter-brain synchrony value for a given task. The black dashed horizontal line represents zero change from pre-baseline.

## Figure S6F: Synchrony in teams vs. individuals at End-baseline (per frequency)

**
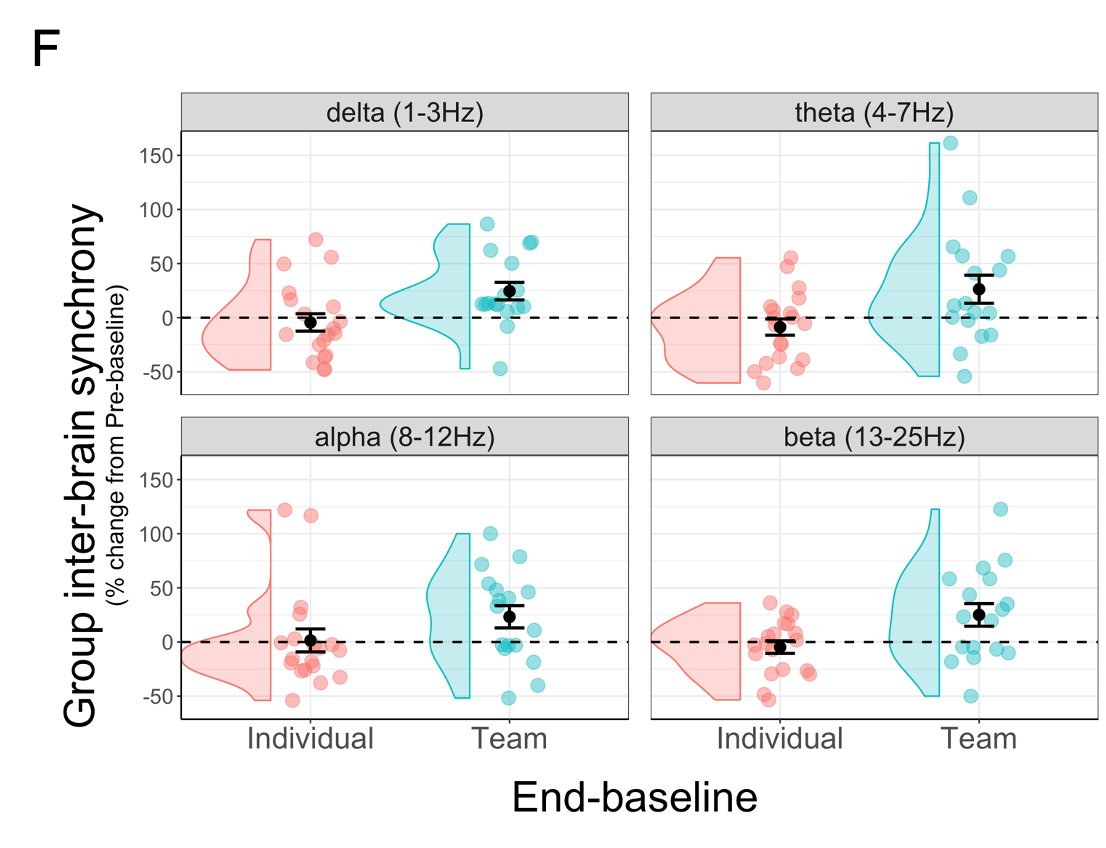
**

The decrease over time in group inter-brain synchrony seen in Figure 6B was slightly buffered for teams (turquoise) and by the end-baseline teams had greater inter-brain synchrony than individuals (red) across all frequency bands. All values represent a group’s percent coherence change from pre-baseline, with each dot representing a group inter-brain synchrony value for a given task. The black dashed horizontal line represents zero change from pre-baseline.

## Figure S7A: Performance ~ Synchrony (max-min norm)


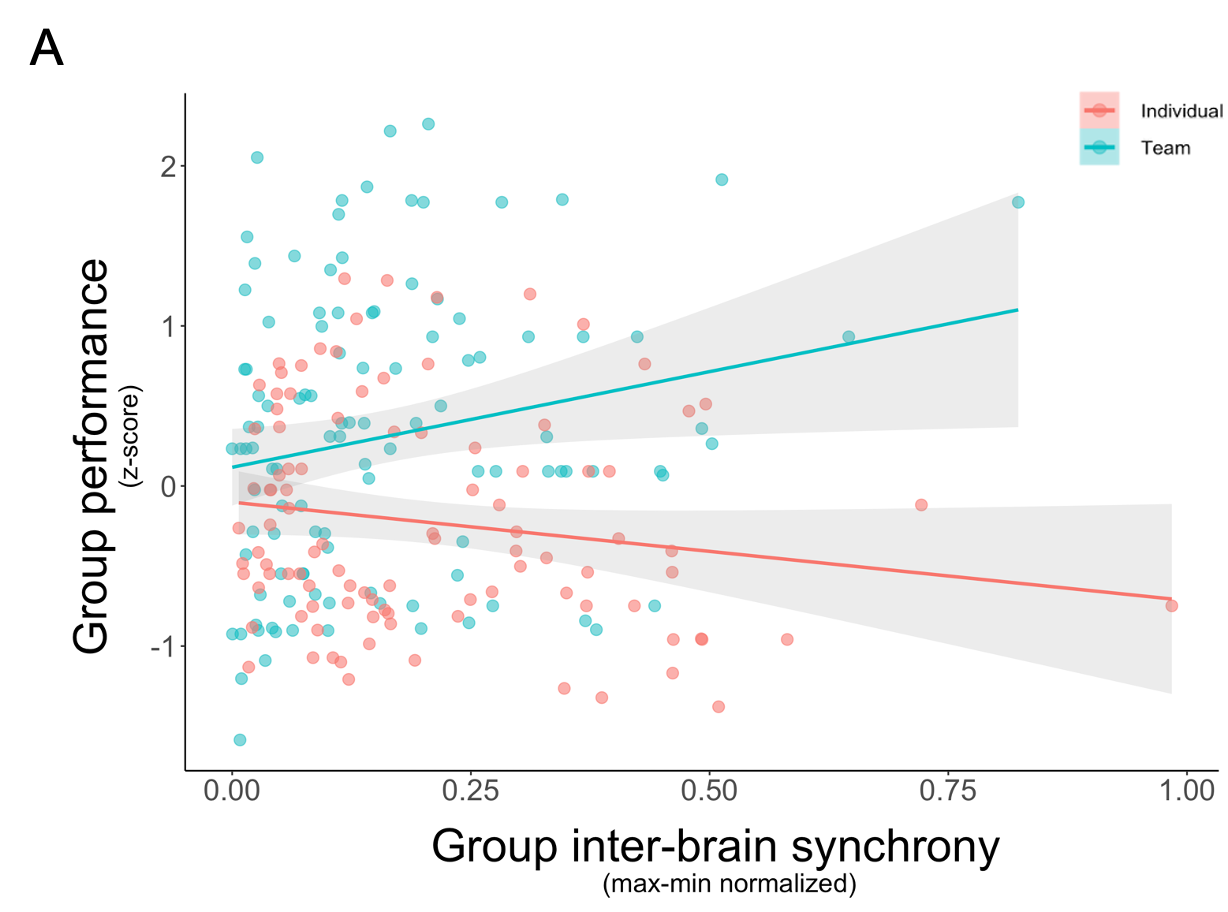


Group inter-brain synchrony predicted performance for teams (turquoise) but not for individuals (red). Synchrony values are max-min normalized and each dot represents a group and a given task. Shading represents 95% confidence interval.

## Figure S7B: Performance ~ Synchrony (per frequency)


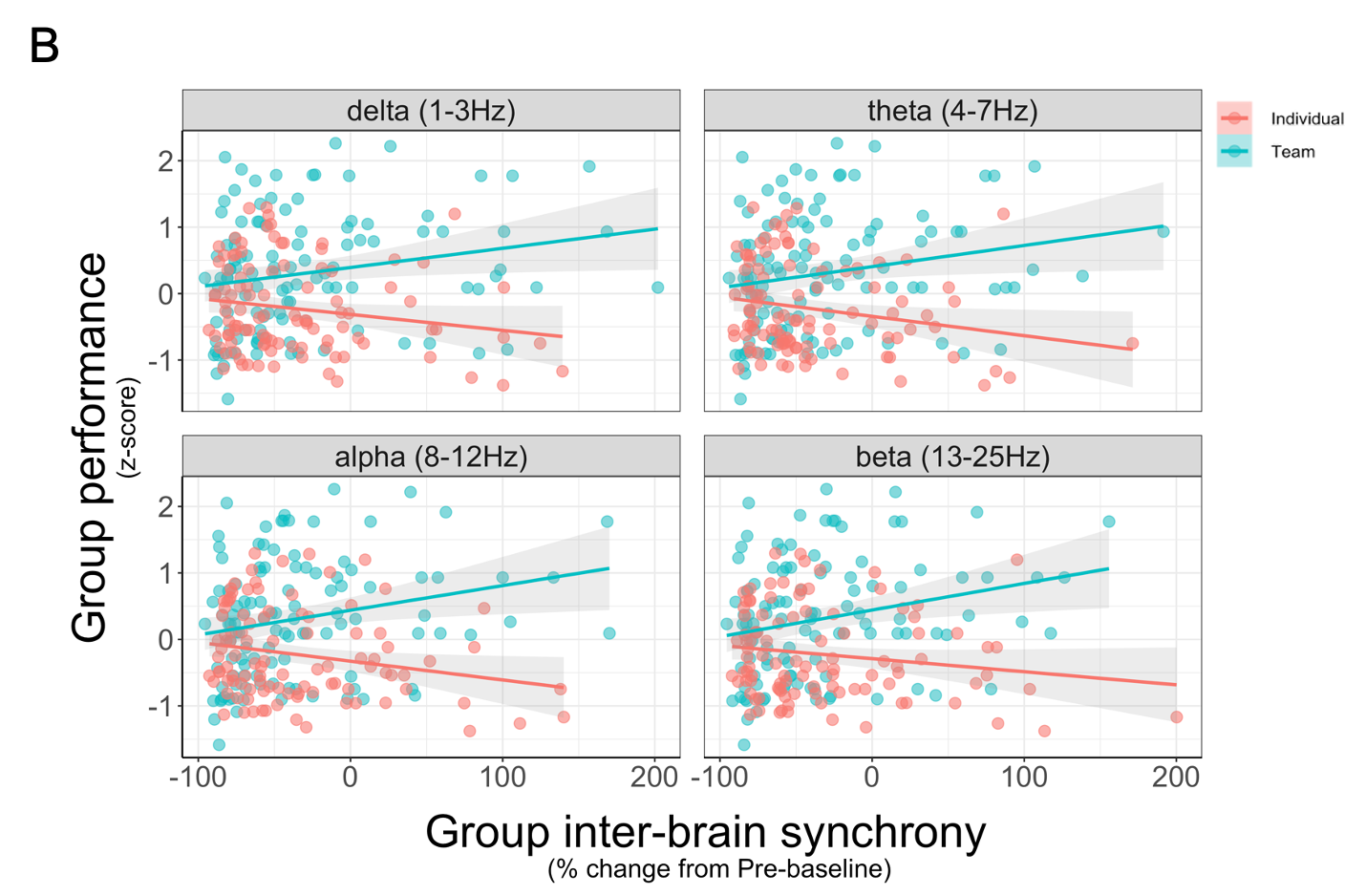


Group inter-brain synchrony predicted performance for teams (turquoise) but not for individuals (red) across all frequency bands. Synchrony values represent each group’s percent coherence change from baseline (the pre-baseline task), and each dot represents a group and a given task. Shading represents 95% confidence interval.

## Figure S7C: Performance ~ Synchrony (per task)


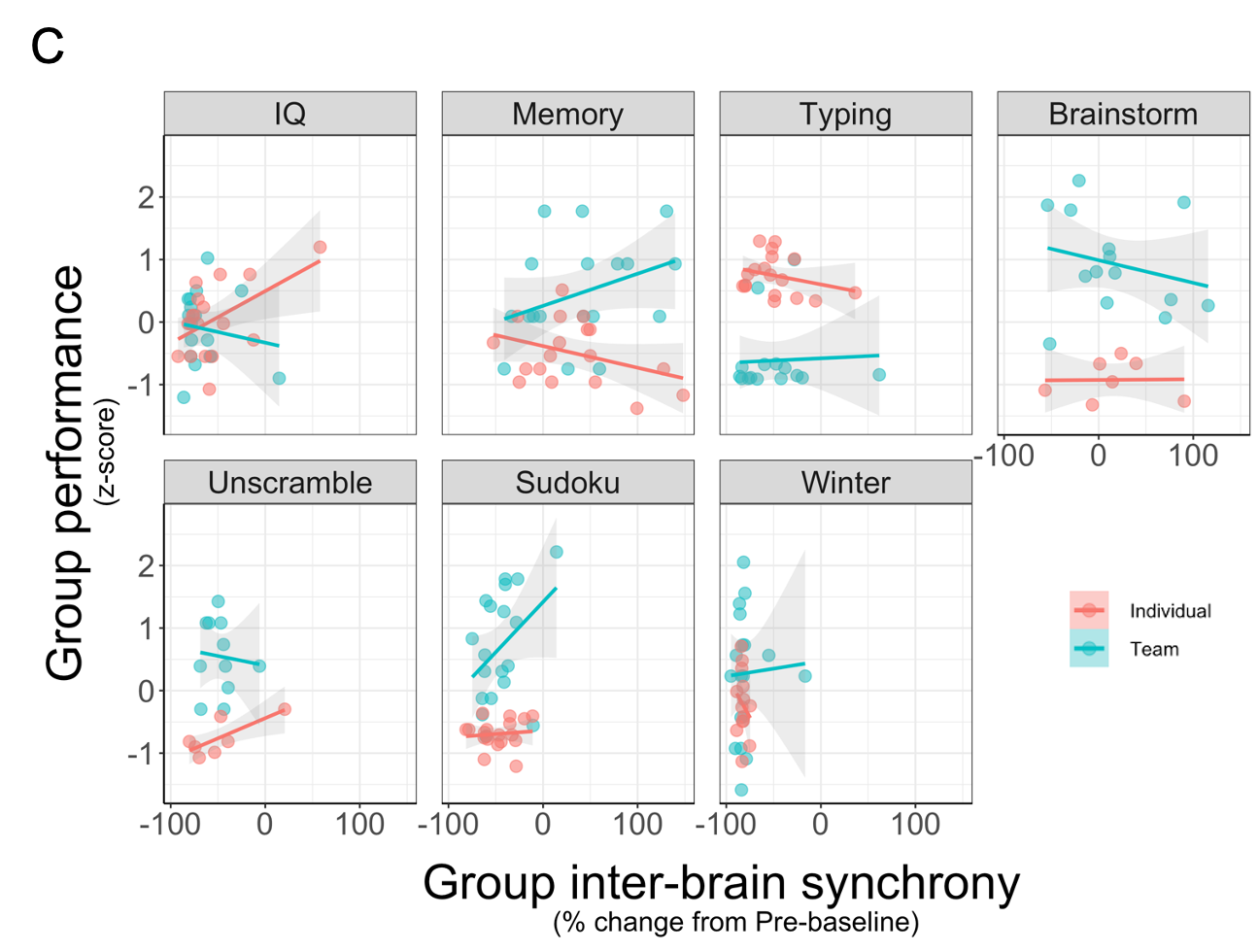


When splitting by task, group inter-brain synchrony did not consistently predict performance for teams (turquoise). However, these sub-task analyses may be underpowered only has an average of 15 group synchrony data points per condition (attrition due to EEG data that was excluded during pre-processing, limiting the number of group inter-brain synchrony values available for a given task). Each dot represents a group and a given task. Shading represents 95% confidence interval.

Exploratory analyses examining the relationship between inter-brain synchrony and performance as a function of each task were mixed (see Figure S7C). It is unclear if these results reflect differences in the constructs measured by each task or the fact that these tests may be underpowered as each task only has an average of approximately 15 group synchrony data points per condition. Thus, although we find that inter-brain synchrony predicts collective performance among teams when incorporating all of our data (and when removing the process-loss task), splitting apart our data by specific tasks yields mixed results. As the signal-to-noise ratio may be low, we are weary of interpreting inter-brain synchrony data on an individual task level.

1. Informally, the Levenshtein distance between two words is the minimum number of single-character edits (i.e. insertions, deletions, or substitutions) required to change one word into the other. [↑](#footnote-ref-1)
